# Evolutionary Pro‐To‐Thr Mutation in the Intrinsically Disordered Domain of ANP32 Family Members Modulates Their Target Binding Modes

**DOI:** 10.1002/advs.202415566

**Published:** 2025-01-31

**Authors:** Blanca Baños‐Jaime, Ana B. Uceda‐Mayo, Francisco Rivero‐Rodríguez, Miguel Á. Casado‐Combreras, Alejandro Velázquez‐Cruz, Adrián Velázquez‐Campoy, Laura Corrales‐Guerrero, Miguel A. De la Rosa, Irene Díaz‐Moreno

**Affiliations:** ^1^ Institute for Chemical Research (IIQ) Scientific Research Center “Isla de la Cartuja” (cicCartuja) University of Seville‐CSIC Avda. Americo Vespucio 49 Seville 41092 Spain; ^2^ Institute for Biocomputation and Physic of Complex Systems (BIFI), Joint Unit GBsC‐CSIC‐BIFI University of Zaragoza C. Mariano Esquillor Zaragoza 50018 Spain; ^3^ Departament of Biochemistry and Molecular and Cellular Biology University of Zaragoza C. Miguel Servet 177 Zaragoza 50013 Spain; ^4^ Institute for Health Research of Aragón (IIS Aragon) Avda. San Juan Bosco 13 Zaragoza 50009 Spain; ^5^ Centre for Biomedical Research Network of Hepatic and Digestive Diseases (CIBERehd) Av. Monforte de Lemos 3–5 Madrid 28029 Spain; ^6^ Present address: Institute of Plant Biochemistry and Photosynthesis (IBVF) Scientific Research Center “Isla de la Cartuja” (cicCartuja) University of Seville‐CSIC Avda. Americo Vespucio 49 Seville 41092 Spain

**Keywords:** cytochrome *c*, HuR, intrinsically disordered domain, isothermal calorimetry, nuclear magnetic resonance

## Abstract

Gene duplication has allowed protein evolution toward novel functions and mechanisms. The differences between paralogous genes frequently rely on the sequence of disordered regions. For instance, in mammals, the chaperones ANP32A and ANP32B share a common evolutionary line and have some exchangeable functions based on their similar N‐terminal domains. Nevertheless, their C‐terminal low‐complexity‐acidic‐regions (LCARs) display substantial sequence differences, unveiling some degree of variability between them, in agreement with their different tissue‐specific expression patterns. These structural and computational results indicate that a substitution in the vicinity of the nuclear localization signal (NLS), of Pro in ANP32A for Thr in ANP32B, determines the overall compactness of the C‐terminal LCAR. The different structural properties of the disordered region affect the binding mode of ANP32 members to their targets. This type of divergent binding mode is exemplified with the extra‐mitochondrial cytochrome *c* (C*c*), a well‐known ANP32B partner and which now determine also binds to ANP32A; and with the RNA binding protein HuR, whose export to the cytoplasm is mediated by ANP32 proteins under stress. Therefore, differential expression patterns of ANP32A or ANP32B may affect the regulation of C*c* and HuR and can help to explain the distinct roles of these proteins in diseases.

## Graphical abstract

Protein evolution usually lies on mutations at disordered domains to promote novel functions and regulations. Here it is demonstrated that this is the case of ANP32A and ANP32B, two paralogs with apparently similar structures and functions, where a single amino‐acid substitution in the disordered domain triggers the extended‐to‐compact conformational change of the unstructured region, causing changes in the recognition processes with different interacting partners.

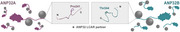

## 1 Introduction

**Figure 1:**
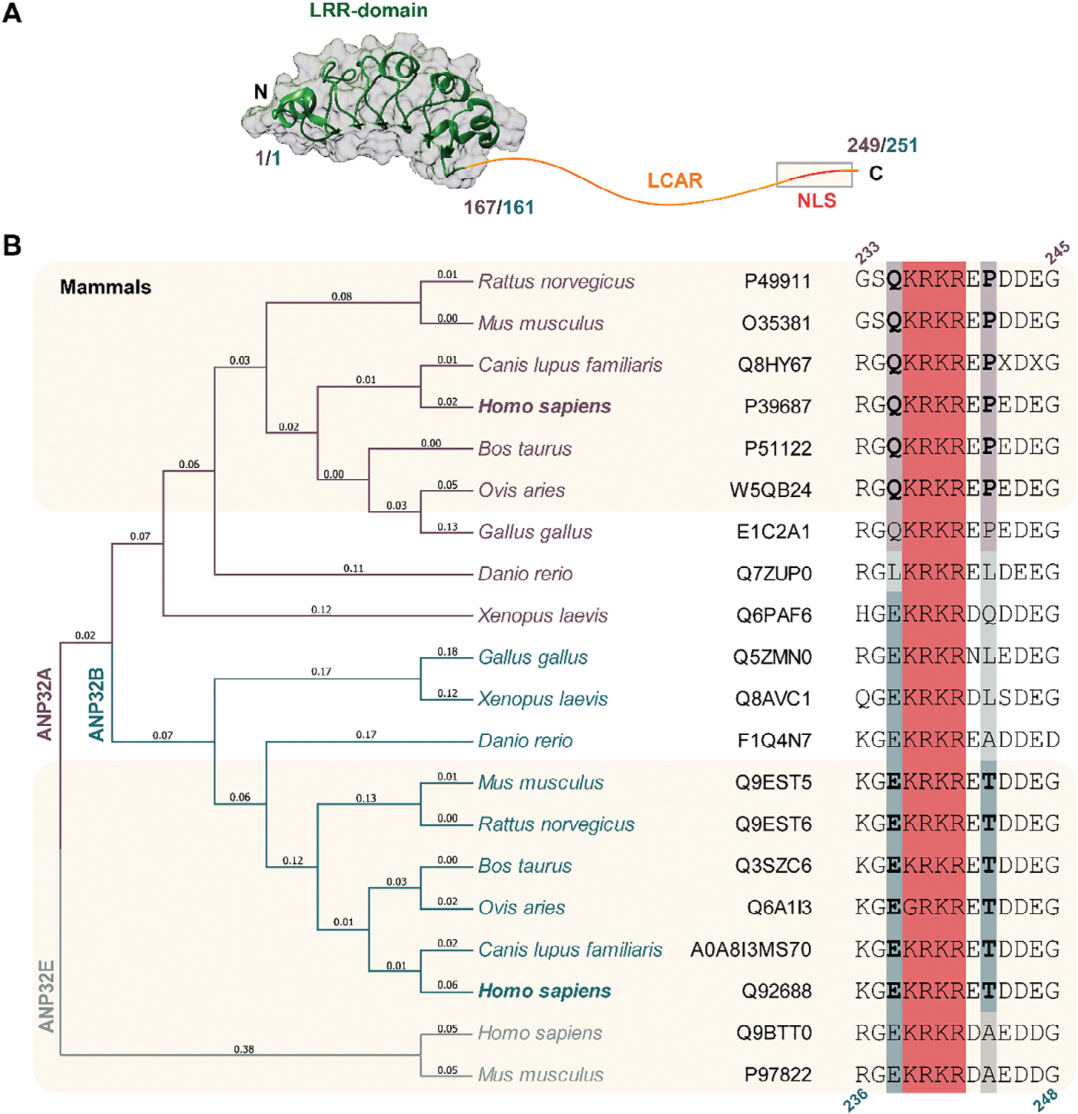
ANP32 protein family structure and alignment. A) Schematic representation of ANP32 protein family domain architecture. The LRR of the ANP32 protein family is shown as a ribbon representation using the X‐ray diffraction model of ANP32A LRR domain (PDB ID: 4XOS), whereas the LCAR is represented as an orange curvy line. The inset indicates the localization of the NLS. Residue numbers corresponding to ANP32A or ANP32B are colored in violet or blue respectively. B) Sequence alignment of the NLS nearby sequence of ANP32 family members from human and related organisms using MUSCLE.^[^ ^36^ ^]^ Both the tree and the organisms are colored in violet, blue or grey according to the indicated protein. Uniprot code for each protein are indicated in the figure. Residues belonging to the NLS are highlighted in pink. The highly conserved residues Gln235 and Pro241 of ANP32A are shaded in violet, whereas Glu237 and Thr244 of ANP32B are in blue. For mammals, Gln235 and Pro241 of ANP32A and Glu237 and Thr244 of ANP32B are in bold letters. Residue numbers corresponding to ANP32A are colored in violet, and to ANP32B, in blue.

The acidic leucine‐rich nuclear phosphoprotein 32 (ANP32) family comprises evolutionary conserved histone chaperones that are present in different organisms of the animal kingdom, with homologous proteins in plants and protists.^[^ ^1^ ^]^ All ANP32 proteins share a common domain arrangement, with a structured N‐terminal leucine‐rich region (LRR) and a C‐terminal intrinsically disordered domain (IDD) (**Figure** **1**A). This IDD is called low‐complexity‐acidic‐region (LCAR), as it contains highly repetitive acidic stretches with no remarkable pattern.^[^ ^2^ ^]^

The human ANP32 family consists of eight members (A to H), of which the proteins A and B are highly conserved (with 71% sequence identity and 81% similarity between the two human proteins).^[^ ^2^, ^3^ ^]^ ANP32B is derived from a gene duplication of ANP32A that evolved independently during the early origin of vertebrates.^[^ ^1^, ^2^ ^]^ This kind of diversification after gene duplication often relies on structurally disordered regions of the proteins.^[^ ^4^, ^5^, ^6^ ^]^ In some cases, retention of gene duplicates—paralogs—is associated with tissue expression divergence.^[^ ^7^, ^8^, ^9^ ^]^ Indeed, ANP32A and ANP32B exhibit a differential expression pattern: while both members are synthesized in brain, placenta, and pancreas, only ANP32A is present in kidney and skeletal muscle, and only ANP32B in heart, lungs, and thymus.^[^ ^10^, ^11^, ^12^ ^]^

Besides their role in the transcription regulation derived from their histone chaperone activity,^[^ ^13^, ^14^, ^15^, ^16^ ^]^ ANP32A and ANP32B share multiple functions, for instance in the RNA metabolism of viruses, such as influenza or human immunodeficiency virus (HIV).^[^ ^17^, ^18^, ^19^, ^20^, ^21^ ^]^ They are also responsible for the nucleo‐cytoplasmic transport of the oncoprotein human antigen R (HuR) upon heat shock. Under regular conditions, ANP32A and ANP32B interact with the RNA‐binding protein (RBP) HuR in the nucleus, and HuR shuttles to the cytoplasm through its shuttling sequence.^[^ ^3^ ^]^ Stress induced by heat shock increases the interaction of HuR with ANP32A and ANP32B and enables HuR to be exported via the nuclear export factor CRM1, which is also responsible for ANP32 protein transport. Moreover, the same domain of HuR that regulates its interactions with the LCAR of ANP32 family members hosts the HuR nucleo‐cytoplasmic sequence (HNS) for HuR transport.^[^ ^22^ ^]^ Further, ANP32A, along with the histone chaperone SET/TAF‐Iβ,^[^ ^23^, ^24^, ^25^, ^26^, ^27^, ^28^, ^29^ ^]^ is a well‐known inhibitor of protein phosphatase 2A (PP2A), whose RNA is, in turn, encoded by RBPs as KH‐Splicing Regulator Protein (KSRP).^[^ ^30^ ^]^ We recently reported that ANP32B is also a PP2A inhibitor, regulated by the extra‐mitochondrial hemeprotein C*c*.^[^ ^31^ ^]^ C*c* migrates from the mitochondria to the cell nucleus under DNA damage conditions.^[^ ^32^, ^33^, ^34^ ^]^ In the nucleus, C*c* targets the disordered C‐terminal LCAR of ANP32B, inducing long‐range allosteric movements into the structured N‐terminal LRR of the histone chaperone and thereby hampering PP2A inhibition.^[^ ^31^ ^]^

Despite the high degree of conservation and the similar functions between ANP32A and ANP32B, the differential tissue expression and the distinct evolution of these proteins suggest that they have (at least in part) distinct cellular functions. This work aims to explain how small differences in an IDD can affect the whole protein dynamics and function. In particular, we demonstrate that the presence of a Pro residue in the LCAR of ANP32A induces a more open and binding‐favorable conformation as compared to its ANP32B counterpart. This Pro‐to‐Thr substitution affects the binding of ANP32 family members with other proteins, such as C*c* and HuR, as evidenced by the point mutation of P241T in ANP32A peptide. Moreover, the ANP32A complexes with C*c* and HuR behave as structurally heterogeneous ensembles (so‐called fuzzy complexes), allowing a high degree of plasticity. Overall, our results suggest distinct regulatory patterns for ANP32A and ANP32B based on their C‐terminal LCAR conformational arrangements.

## 2 Results

### 2.1 Pro‐To‐Thr Substitution Alters the Overall Structure and Dynamics of ANP32 LCAR

All the ANP32 family members except C and D contain a NLS in the C‐terminus of the LCAR, the most variable region between ANP32A and ANP32B in sequence, besides the fact that ANP32A LCAR has a higher proportion of aromatic residues. The functional relevance of the C‐terminal LCAR region has been recently highlighted, since the deletion of the last 20 residues in ANP32B was enough to alter the thermodynamics of the binding to C*c* leading to differences in its functionality.^[^ ^31^ ^]^ The NLS stands out because it contains numerous basic residues, and in particular Lys and Arg,^[^ ^1^, ^35^ ^]^ in contrast to the highly acidic nature of the remaining LCAR. Thus, the mobility of the C‐terminal region may be affected by electrostatic attractions between the NLS and the negative residues along the LCAR, with a plausible impact on protein‐protein interactions involving the LCAR. Therefore, these structural differences between LCARs could represent a reason for the functional variations between ANP32 family members. To understand the evolution of the ANP32 family LCAR, we aligned the sequences of ANP32 family members A, B, and E in diverse organisms, with a special focus on the NLS nearby sequence (Figure 1B). Although some of the residues surrounding the NLS vary according to the organisms, we found two highly conserved residues in mammals: Gln235 and Pro241 in human ANP32A, and the equivalent residues of Glu237 and Thr244 in human ANP32B, respectively. The highly conserved changes in ANP32A and ANP32B in the evolutionary branch where mammals arose suggest a specialization of each ANP32 protein. Moreover, given the conformational restraint of Pro residues, we wondered if the Pro‐to‐Thr substitution might modify the dynamic properties of the C‐terminal region of the LCAR, thus affecting the function and/or regulation of ANP32B.

**Figure 2:**
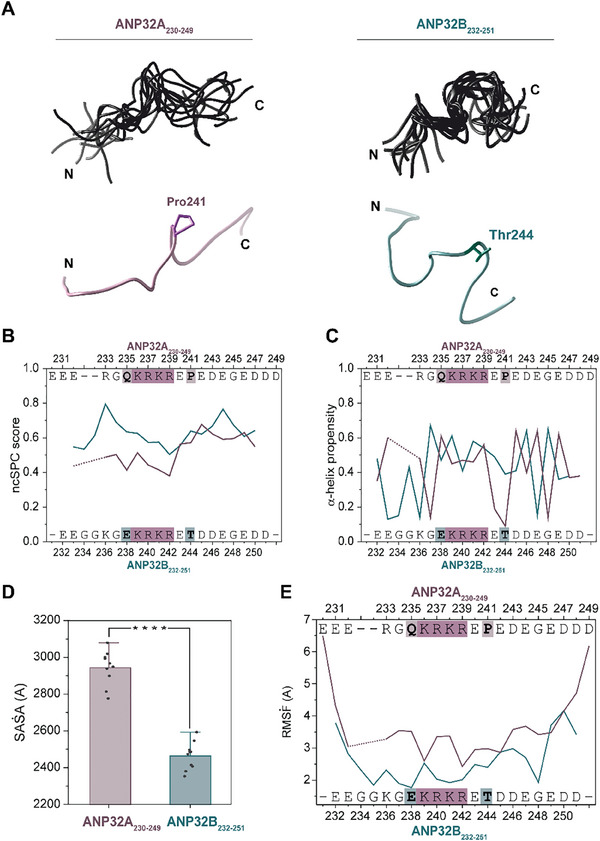
Structural models of ANP32A_230‐249_ and ANP32B_232‐251_ peptides. A) Ribbon representations for the structural models of ANP32A_230‐249_ (*left*) and ANP32B_232‐251_ peptides (*right*). *Upper panels* show the overlapping of the top ten models calculated by CYANA for each construct. *Lower panels* show the #0.1 solution provided by CYANA. Pro241 is highlighted in pink in the ANP32A_230‐249_ peptide model, and Thr244, in blue in the ANP32B_232‐251_ peptide model. B) Residue‐specific Neighbor Corrected Structural Propensity Calculator (ncSPC) scores obtained for ANP32A_230‐249_ and ANP32B_232‐251_ peptides. “+1″ indicates the maximum propensity to form a full α‐helix, and “0” indicates disorder. C) α‐helix propensity scores obtained for ANP32A_230‐249_ and ANP32B_232‐251_ peptides. The values were obtained using the CSI3.0 web server. D) Mean of the solvent‐accessible surface area (SASA) of the ten selected structures of ANP32A_230‐249_ and ANP32B_232‐251_ peptides. E) Atomic fluctuations (Root mean square fluctuation, RMSF) values of backbone atoms between the ten selected structures of each peptide as a function of residue number. ANP32A_230‐249_ data are represented in violet, and ANP32B_232‐251_ data, in blue. Residues of each peptide are indicated in graphs B, C, and E. Residues belonging to the NLS are highlighted in pink. Gln235 and Pro241 of ANP32A are shaded in violet, whereas Glu237 and Thr244 of ANP32B are in blue.

To address these questions, we designed synthetic peptides containing the last 20 amino acids of the ANP32A or ANP32B human protein (ANP32A_230‐249_ and ANP32B_232‐251_). Structural features of these peptides were studied in detail by Nuclear Magnetic Resonance (NMR) 2D ^1^H‐^1^H Total Correlation Spectroscopy (TOCSY) and nuclear Overhauser effect spectroscopy (NOESY) (Figure S1, Supporting Information). Using NOEs corresponding to the C‐end NLS‐containing stretch of ANP32A or ANP32B, we obtained model ensembles for the peptides comprising the ten structures with the lowest energy values. Structure calculations with CYANA included the interatomic/inter‐residue distances inferred from NOESY cross‐correlation peak intensities as restraints. Nevertheless, such structural models partially represent the total conformational landscape of each peptide, including those with a preferential pattern of contacts among residues. The results obtained after structural refinement–consisting of several rounds of distance violation analysis and filtering–showed that both peptides substantially differed in their conformational arrangements (**Figure** **2**A). Specifically, the ANP32A_230‐249_ peptide exhibited a broader, more diverse ensemble of conformations, with a higher backbone root‐mean‐square deviation (RMSD) between the ten selected models with respect to that of the ANP32B_232‐251_ peptide (Table S1, Supporting Information). In addition, the ANP32A_230‐249_ peptide showed open, extended conformations, in which Pro241 keeps the N‐ and C‐ends separated from each other (Figure 2A). In contrast, the ANP32B_232‐251_ peptide predominantly adopted closed, compact conformations, likely driven by the formation of Thr‐enhanced transient contacts between the acidic N‐ and C‐ ends with the basic NLS stretch. Moreover, the ANP32B_232‐251_ peptide exhibited a larger tendency for α‐helix formation than ANP32A_230‐249_ peptide, supporting the idea of a more compact and best‐defined conformation for ANP32B LCAR (Figure 2B,C).

We further analyzed the solvent‐accessible surface area (SASA) of the top 10 lowest‐energy conformers of each peptide, as well as their atomic fluctuations (root‐mean‐square‐fluctuation, RMSF) (Figure 2D,E). ANP32A_230‐249_ exhibited a significantly higher exposure to solvent than ANP32B_232‐251_. Moreover, the ANP32B_232‐251_ peptide showed a decreased inter‐model RMSF as compared to ANP32A_230‐249_, in agreement with the lower backbone RMSD values and higher compaction degree inferred from CYANA‐calculated NMR models. Altogether, our NMR structural models revealed that the ANP32A_230‐249_ stretch mainly adopts open conformations with a large global mobility, as inferred from backbone RMSD and RMSF values in addition to solvent accessibility, whereas the ANP32B paralog mainly adopts closed conformations.

A different conformation propensity of the LCAR could affect the manner in which ANP32 proteins interact with their different binding partners. Therefore, we first compared the binding capacity of ANP32A and ANP32B to histones. To test that, we performed a coimmunoprecipitation experiment after transfection of HeLa cells with expression vectors for *c*‐myc‐tagged ANP32A or ANP32B. The presence of histone H3 in the immunoprecipitated sample with anti‐*c*‐myc, detected by western blot, confirmed that H3 binds both ANP32 proteins in a similar manner (Figure S2A, Supporting Information). Thus, LCAR differences do not affect histone binding of ANP32 proteins, in agreement with that being mediated by the LRR domains.^[^ ^14^ ^]^

### 2.2 The LCAR of ANP32A Interacts with Cytochrome *c* Under DNA Damage Conditions

**Figure 3:**
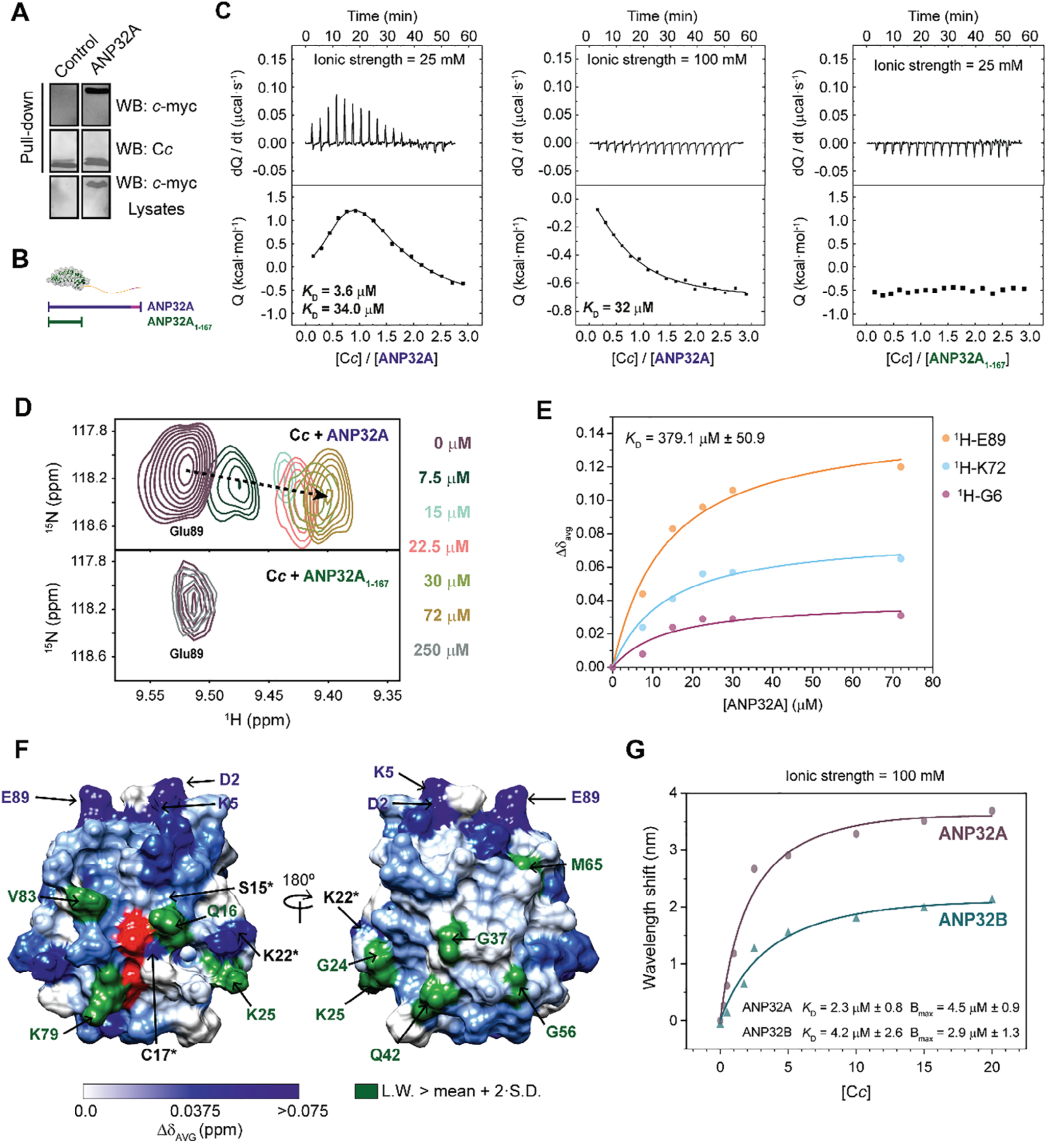
ANP32A interaction with C*c*. A) *Upper panel*: Western blot against *c*‐myc tag, at the C‐terminus of ANP32A, after performing a pulldown assay with extracts from cells transfected with either the empty vector (control) or the pCDNA3.1‐ANP32A‐c‐myc. *Middle panel*: Western blot against C*c* demonstrating that the hemeprotein was captured within the carboxymethyl cellulose matrix. *Lower panel*: Western blot against *c*‐myc tag of HEK293T lysates as a loading and transfection control. B) Schematic representation of the ANP32A and ANP32A_1‐167_ constructs, used in D and E. C) ITC titrations of ANP32A with C*c* at low (*left panel*) and moderate (*center panel*) ionic strength; and of ANP32A_1‐167_ and C*c* at low ionic strength (*right panel*). Thermograms and binding isotherms are shown in the *upper* and *lower* panels, respectively. D) Detailed view of the superimposed 2D ^1^H‐^15^N HSQC spectra of ^15^N‐labeled C*c* during titrations with increasing concentrations of ANP32A (*upper panel*) or ANP32A_1‐167_ (*lower panel*) at low ionic strength. The color guide of ANP32A constructs concentration is shown in the panel. E) Curves representing the best global fit of several amide signals in the direct (^1^H) dimension with a global *K*_D_ value for ANP32A binding with C*c*. F) Representation of averaged chemical‐shift perturbations (Δδ_AVG_) and broadening of C*c* amide signals upon C*c*‐ANP32A complex formation at a 1:1 ratio (C*c*:ANP32A). Δδ_AVG_ is represented by a blue color gradient, ranging from white (Δδ_AVG_ = 0.0 ppm) to dark blue (Δδ_AVG_ > 0.075 ppm). Residues exhibiting Δδ_AVG_ > 0.075 ppm are marked with blue labels. Residues showing a line‐broadening larger than the average plus two SDs (15.96 Hz) are in green. Residues labeled with an asterisk show both significant Δδ_AVG_ and broadening. The heme group is in red. G) Biolayer Interferometry analysis of the interaction between ANP32A or ANP32B with C*c*. The wavelength shifts at equilibrium were plotted against C*c* concentration.

To determine if the Pro/Thr and/or the Gln/Glu substitution could enable binding discrimination through the LCAR region, we tested as a binding partner the hemeprotein C*c*, which specifically recognizes the ANP32B C‐terminal NLS‐containing stretch.^[^ ^31^ ^]^ We first tested whether C*c* can also recognize the ANP32A LCAR by a pulldown assay, using extracts from HEK293T cells transfected with either the empty pCDNA3.1 vector or that encoding ANP32A‐*c*‐myc, and incubated with recombinant C*c*. The resulting C*c*:ANP32A complex was co‐purified, revealing that C*c* interacts with ANP32A (**Figure** **3**A). After treating Heltog cells (HeLa cells constitutively expressing the C*c* gene fused to green fluorescent protein [GFP])^[^ ^37^ ^]^ with camptothecin (CPT) for 1 or 4 h, to induce DNA double‐strand breaks,^[^ ^38^ ^]^ we observed that C*c*‐GFP translocated into the cell nucleus, where also localizes ANP32A (Figure S2B, Supporting Information). Colocalization of ANP32A and C*c* in the cell nucleus under DNA damage conditions supports the possible interaction between these proteins in the cellular context, as it was previously demonstrated for ANP32B.^[^ ^31^, ^39^ ^]^

Due to the high sequence similarity between ANP32A and ANP32B, it seems plausible that C*c* recognizes both proteins at the same region. Since the interaction of the LCAR of ANP32B with C*c* is well established,^[^ ^31^ ^]^ we performed Isothermal Titration Calorimetry (ITC) assays with C*c* and either the full‐length ANP32A or a construct lacking its LCAR (ANP32A_1‐167_) (Figure 3B). ITC analyses showed that ANP32A binds to C*c* at both low (25 mM) and moderate (100 mM) ionic strength, although one of the two binding sites was lost in the latter case (Figure 3C). Interestingly, ANP32A_1‐167_ was unable to interact with C*c* even at low ionic strength. This result suggests that C*c* exclusively recognizes the LCAR of ANP32A. Titration of ANP32A with C*c* at low ionic strength indicated that two molecules of C*c* bind the ANP32A LCAR at independent binding sites, with no observed cooperativity (**Table** **1**). Notably, C*c* binding to ANP32A‐LCAR is entropically driven, similar to many other complexes involving IDDs.^[^ ^40^, ^41^, ^42^ ^]^ At moderate ionic strength, data are consistent with a single site for C*c* with low affinity (ca. 32 µM) and have enthalpic and entropic values similar to those obtained at low ionic strength.

**Table 1:** Thermodynamic parameters of the cytochrome *c*:ANP32A complex at low or moderate ionic strength.

Ionic strength [mM]	*K*_D_ [µM]	Δ*G* [kcal mol^−1^]	Δ*H* [kcal mol^−1^]	–*T*Δ*S* [kcal mol^−1^]	*n*
25	3.6	−7.4	0.4	−7.8	0.65
	34.0	−6.1	8.6	−14.1	0.65
100	32.0	−6.1	1.5	−7.6	0.60

Thermodynamic parameters for the interaction of ANP32A with C*c* at low and moderate ionic strength. Enthalpy (Δ*H*), entropic contribution (–*T*Δ*S*), equilibrium dissociation constant (*K*_D_), Gibbs free energy (Δ*G*), and reaction stoichiometry (*n*) are shown. Relative errors: *K*_D_, 20%; Δ*G*, 0.1 kcal/mol; Δ*H* and –*T*Δ*S*, 0.4 kcal/mol; and *n*, 0.03.

Additionally, we recorded 2D ^1^H‐^15^N heteronuclear single quantum correlation (HSQC) spectra of ^15^N C*c*, either free or in the presence of increasing amounts of ^14^N full‐length ANP32A or ANP32A_1‐167_, under low ionic strength conditions. C*c* amide signals showed no significant chemical‐shift perturbations in titrations with ANP32A_1‐167_, further corroborating the ITC findings (Figure 3D). A thorough analysis of the C*c*:ANP32A complex at a 1:1 ratio shows that particular C*c* resonances experience specific chemical‐shift perturbations and substantial broadening (Figure S3, Supporting Information), consistent with an intermediate chemical exchange rate within the NMR timescale and in agreement with the transitory nature of the complex and its dissociation constant (*K*_D_) within the micromolar range (Table 1 and Figure 3E).

To delimit the interaction surface between C*c* and ANP32A, we represented the averaged chemical‐shift perturbations (Δδ_AVG_) and linewidth broadening of C*c* amide resonances on the C*c* surface (Figure 3F). Most residues with large Δδ_AVG_ and significant broadening map close to the heme crevice. In addition, an additional cluster of perturbed residues with either significant Δδ_AVG_ or broadening are located at the opposite face of the C*c* heme group. This spread pattern of interacting residues supports the formation of structurally heterogeneous fuzzy ensembles, in which the chaperone´s LCAR plays a crucial role.^[^ ^43^, ^44^, ^45^ ^]^ Thus, the ANP32A LCAR is likely represented by an ensemble of exchanging conformations, which in turn facilitate its interaction with C*c*.

### 2.3 Cytochrome *c* Differentiates Between the LCARs of ANP32A and ANP32B

Although both ANP32A and ANP32B interact with C*c* via their C‐terminal region, there are remarkable differences in the thermodynamic parameters and binding surfaces between full‐length ANP32A and ANP32B in complex with C*c* (Figure 3C,F).^[^ ^31^ ^]^ At low ionic strength, C*c* binds to ANP32A with higher affinity (*K*_D_ ca. 3.6 µM) than the one previously reported for ANP32B (*K*_D_ ca. 9.5 µM). Strikingly, the ANP32A and ANP32B interactions with C*c* present very different thermodynamic profiles (C*c*‐ANP32A, ΔH: 0.4 kcal mol^−1^ and ‐TΔS: ‐7.8 kcal mol^−1^; C*c*‐ANP32B, ΔH: ‐10.7 kcal mol^−1^ and ‐TΔS: 3.9 kcal mol^−1^; Table 1).^[^ ^31^ ^]^ Moreover, ANP32A contacts C*c* through an extended surface. Therefore, C*c* likely presents different modes of recognizing the LCAR of both chaperones. To confirm the differences in binding, we performed Biolayer Interferometry assays by attaching biotinylated ANP32 proteins to a streptavidin‐coated sensor and incubating with increasing concentrations of C*c* (Figure 3G). Again, C*c* interacted with ANP32A with higher affinity than with ANP32B (C*c*‐ANP32A, *K*_D_ ca. 2.3 µM; C*c*‐ANP32B, *K*_D_ ca. 4.2 µM). Also, the total number of C*c* molecules that interact with the sensor (B_max_) was greater for C*c*‐ANP32A complex, in consonance with the broader interaction surface.

To corroborate our hypothesis that the divergent conformations and dynamics of the NLS‐containing peptides are responsible for the different interactions with protein partners, we scaled‐down the system by performing ITC assays between the NLS‐containing peptides (ANP32A_230‐249_ and ANP32B_232‐251_) and C*c*. This simplification of the binding model facilitated a picture of the thermodynamic profile of C*c* binding to the NLS‐containing regions. ITC‐derived data were fitted to a single binding‐site model (Figure S4, Supporting Information). Both peptides exhibited much higher *K*_D_ values than the full‐length ANP32A/B protein when interacting with C*c*, indicating the relevance of the acidic content of the entire LCAR. The interaction between C*c* and ANP32A_230‐249_ was mainly entropically driven (–TΔS: ‐3.1 kcal mol^−1^; Table 2), as for the full‐length protein. Surprisingly, C*c* presented a 10‐fold higher affinity for ANP32A_230‐249_ than for ANP32B_232‐251_ (**Table** 2); our structural models suggest that this could be due to the ANP32A peptide adopting more appropriate conformations for C*c* binding (Figure 2A).

**Table 2:** Thermodynamic parameters of the cytochrome *c*:ANP32A/B peptide complexes.

Protein complex	*K*_D_ [µM]	Δ*G* [kcal mol^−1^]	Δ*H* [kcal mol^−1^]	−*T*Δ*S* [kcal mol^−1^]	*n*
C*c*:ANP32A_230‐249_	69	−5.7	−2.6	−3.1	0.76
C*c*:ANP32B_232‐251_	710	−4.3	−8.0	3.7	1.20
C*c*:ANP32A_230‐249_ P241T	190	−5.1	−6.9	1.8	0.77
C*c*:ANP32A_230‐249_ Q235E/P241T	190	−5.1	−8.2	3.1	0.77

Thermodynamic parameters for the interactions of ANP32A/B peptides with C*c*. Enthalpy (Δ*H*), entropic contribution (–*T*Δ*S*), equilibrium dissociation constant (*K*_D_), Gibbs free energy (Δ*G*), and reaction stoichiometry (*n*) are shown. Relative errors: *K*_D_, 20%; Δ*G*, 0.1 kcal/mol; Δ*H* and –*T*Δ*S*, 0.4 kcal/mol; and *n*, 0.03.

We then explored the specific contributions of the major amino acid substitutions between the ANP32A and ANP32B peptides (i.e., Gln235 and Pro241) to the binding event. We designed two additional synthetic 20‐residue peptides, using the ANP32A_230‐249_ wild‐type stretch as a template, to suppress differences between the ANP32A and ANP32B LCARs. One of these peptides contains a P241T single mutation, and the other, a Q235E/P241T double mutation. Of note, the single substitution of Pro‐to‐Thr in ANP32A_230‐249_ was necessary and sufficient to: i) weaken the binding between C*c* and the peptide, and ii) cause a loss of conformational entropy and render the binding into an entropically‐driven one (Table 2 and Figure S4, Supporting Information). The double Q235E/P241T ANP32A_230‐249_ mutant reproduced the thermodynamic profile of ANP32B_232‐251_ complexed with C*c* (Table 2 and Figure S4, Supporting Information). Although complex affinity was diminished for both the single and the double mutants, neither showed *K*_D_ values as high as those determined for the C*c*:ANP32B_232‐251_ complex. A plausible explanation is the presence of an additional Gly‐Gly pair in the ANP32B_232‐251_ peptide, which provides a slightly different molecular context and is likely responsible for changes in the global mobility and internal dynamics of the molecule.^[^ ^46^ ^]^

**Figure 4:**
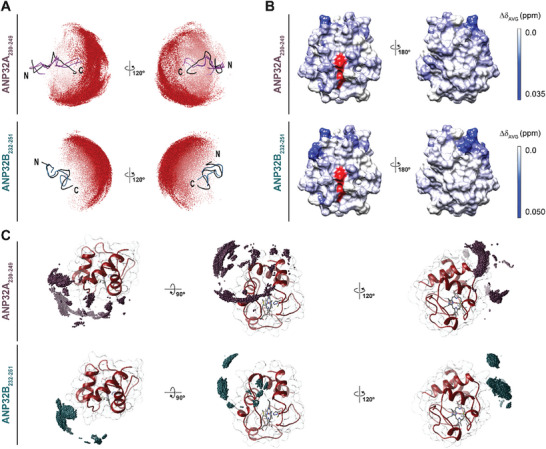
Cytochrome *c* differentiates between the LCAR of ANP32A and ANP32B. A) Simulated flexible *ab‐initio* Brownian dynamics solutions for C*c* sampling of the ANP32A_230‐249_ (*upper panels*) and ANP32B_232‐251_ (*lower panels*) peptides. Representations of the peptide structures show ribbons colored in black for the best‐fitted solution provided by CYANA for each peptide. Mass centers of C*c* (corresponding to the 50000 registered complexes for each simulation) are shown as red spheres. B) Representation of Δδ_AVG_ of C*c* amide signals upon C*c*‐ANP32A_230‐249_ (*upper panel*) or C*c*‐ANP32B_232‐251_ (*lower panel*) complex formation at 1:6 ratio (C*c*:peptide). Δδ_AVG_ is represented as a blue color gradient, ranging from white (Δδ_AVG_ = 0.0 ppm) to dark blue, whereby Δδ_AVG_ > 0.035 ppm or 0.050 ppm for C*c*:ANP32A_230‐249_ and C*c*:ANP32B_232‐251_, respectively. The heme group is represented in red. C) NMR‐driven docking solutions for ANP32A_230‐249_ and ANP32B_232‐251_ complexes with C*c*. Mass centers distributions of ANP32A_230‐249_ (*upper panels*) or ANP32B_232‐251_ (*lower panels*), representing the 10000 lowest energy conformations of both complexes with C*c*. Mass centers are shown as violet spheres for ANP32A_230‐249_ and blue spheres for ANP32B_232‐251_. Ribbon and surface areas of C*c* (PDB entry 2N9I) are shown in red; the heme group, in light green.

To explore the impact of different conformational arrangements of ANP32A_230‐249_ or ANP32B_232‐251_ in binding to C*c*, we performed ab initio flexible Brownian dynamics (BD) simulations. The 10 best conformers of each peptide—inferred from CYANA calculations from NOESY experiments (Figure 2A)—were selected and set as targets of C*c*. C*c* approaching trajectories to the alternating conformations of both paralog peptides were monitored. Our results showed that C*c* preferably explores the C‐end of the ANP32A_230‐249_ or ANP32B_232‐251_ peptide (**Figure** **4**A), where negatively‐charged residues accumulate. However, the area explored by C*c* on the ANP32A_230‐249_ peptide was wider than that on the ANP32B_232‐251_. This finding agrees with the open local conformation of the ANP32A_230‐249_ stretch and its larger SASA, which may facilitate C*c* binding.

NMR titrations of ^15^N‐labeled C*c* with both ANP32A_230‐249_ and ANP32B_232‐251_ peptides showed that the chemical‐shift perturbations spread over the whole surface of C*c*–including the opposite face to the heme group–was induced mainly by the ANP32A_230‐249_ peptide (Figure 4B). To visualize the C*c* surface sampled by both ANP32 C‐terminal peptides, we performed NMR‐driven docking simulations. C*c* was set as the target of the most represented conformation of ANP32A_230‐249_ or ANP32B_232‐251_ peptides among the complexes provided by the aforementioned flexible BD calculations, and experimental restrictions based on NMR titrations (Figure 4B) were imposed. Simulations rendered mass centers for ANP32A_230‐249_ showing a widely distributed sampling of C*c*, from its heme‐surrounding region to its opposite side (Figure 4C), resembling the NMR spectra obtained with full length ANP32A on C*c* (Figure 3F). Such distribution of complexes was narrowed down to a more limited area for the ANP32B_232‐251_ peptide (Figure 4C), in agreement with the map of NMR chemical‐shift perturbations (Figure 4B). This difference is likely due to a more compact conformation of the ANP32B_232‐251_ peptide limiting its ability to sample the hemeprotein. Notably, ab initio BD simulations yielded very similar complex distributions for both interactions (Figure S5, Supporting Information), confirming that the obtained complexes are the most energetically favorable.

**Figure 5:**
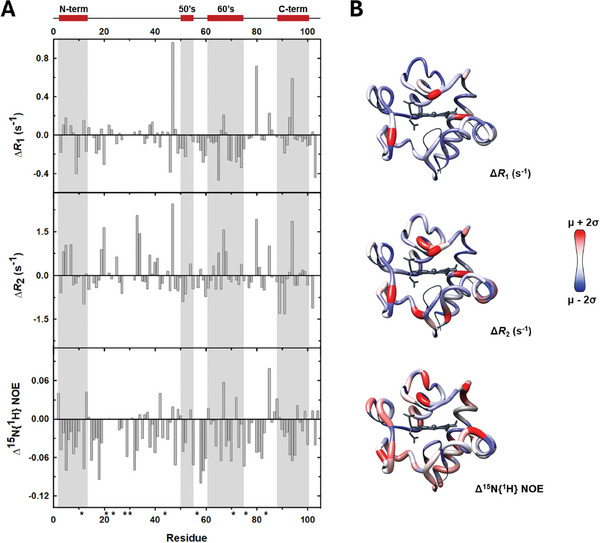
Differences in the dynamics of C*c* in the presence of ANP32 peptides. A) Differences in longitudinal relaxation rate *R*_1_ (*top*), transversal relaxation rate *R*_2_ (*middle*), and heteronuclear ^15^N{^1^H} NOE (*bottom*) between the experimental values at 500 MHz for the complexes formed between the reduced form of C*c* and ANP32A_230‐249_ or ANP32B_232‐251_ peptides, plotted as a function of the residue number. Asterisks (*) indicate prolines and non‐assigned residues. A scheme of the secondary structure elements of C*c* is included at the top. B) Ribbon structure of C*c* (PDB: 2N9I) colored according to the difference in its dynamic properties when forming complexes with ANP32A_230‐249_ (*red*) or ANP32B_232‐251_ (*blue*) peptides. Prolines, non‐assigned residues and the heme group are in grey. Models has been calculated with CHIMERA 1.14.

To further corroborate differences in the dynamic behavior of ^15^N‐C*c*/ANP32A_230‐249_ and ^15^N C*c*/ANP32B_232‐251_ complexes, NMR relaxation measurements were recorded (**Figure** **5**A,B). Binding to ANP32A and ANP32B peptides did not significantly alter the rotational correlational time (τ_c_) of C*c* with respect to free hemeprotein (free C*c* 6.0 ns, ANP32A_230‐249_‐bound C*c* 5.9 ns, ANP32A_232‐251_‐bound C*c* 5.8 ns) likely due to the small molecular weight of both peptides. Comparing the differences in *R*_1_, *R*_2_, and ^15^N{^1^H} NOE parameters revealed that the interface of N‐terminal and C‐terminal α‐helixes in foldon I of ANP32A‐bound C*c* exhibited higher mobility in the ps‐to‐ns timescale, as the ^15^N{^1^H} NOE values for the V3, K5, K8, D93, L94, I95 residues are smaller for ANP32A‐bound C*c* protein. Similar behavior is observed for the most of 60´s helix of C*c* (foldon II) in complex with ANP32A. Altogether, this finding supports the highly dynamic behavior of C*c* upon binding to the ANP32A_230‐249_ peptide, in line with a fuzzy recognition manner. In addition, C*c* residues in complex with ANP32A_230‐249_ experienced larger *R*_2_ rates than in the presence of ANP32B_232‐251,_ due to conformational exchange in the µs‐to‐ms timescale that constraints local motions. The scattered pattern of perturbed residues on the C*c* surface perfectly matches with NMR chemical‐shift perturbation maps and NMR‐restraint docking calculations (Figure 4B,C).

### 2.4 Pro‐To‐Thr Substitution also Affects ANP32 Binding to HuR

**Figure 6:**
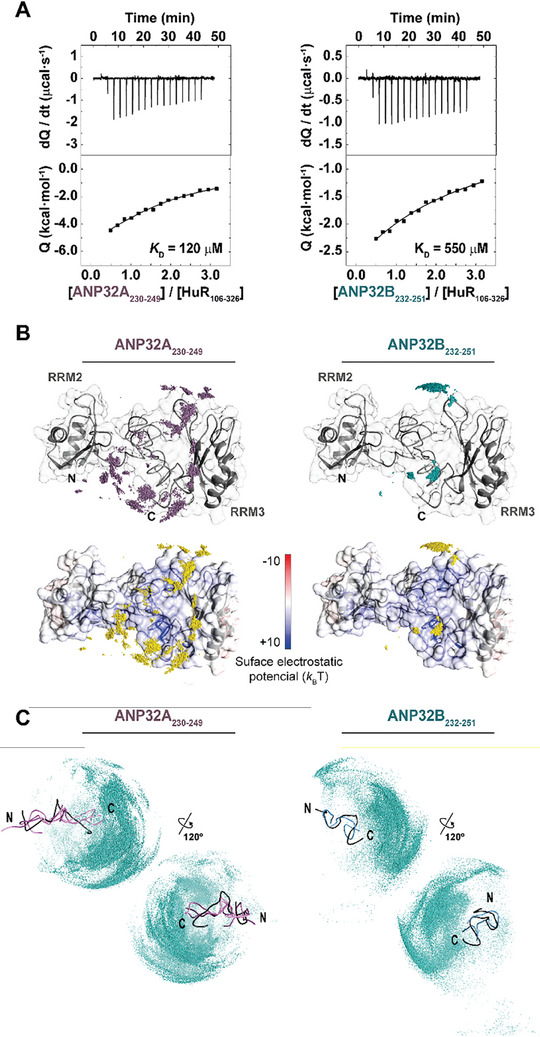
HuR differentiates between the LCAR of ANP32A and ANP32B. A) ITC binding experiments of HuR_106‐326_ with ANP32A_230‐249_ and ANP32B_232‐251_ peptides at low ionic strength. Thermograms and binding isotherms are shown in the *upper* and *lower* panels, respectively. B) Ab initio docking solutions for ANP32A_230‐249_ and ANP32B_232‐251_ complexes with HuR_106‐326_ obtained by Brownian dynamics. Mass centers distributions of ANP32A_230‐249_ (*left panels*) or ANP32B_232‐251_ (*right panels*), representing the 10000 lowest energy conformations of both complexes with HuR_106‐326_. Ribbon colored in gray and surface electrostatic potentials—represented as a gradient from red to blue— of HuR_106‐326_ at 15 mM ionic strength are shown. Mass centers are shown as violet spheres for ANP32A_230‐249_ (*upper‐left*) and blue spheres for ANP32B_232‐251_ (*upper‐right*). In *lower panels*, mass centers of both ANP32 peptides are represented by yellow spheres. The structural model of HuR_106‐326_ to trace the unstructured linker between domains RRM2 and RRM3, was built using as templates X‐ray structures of both (PDB entries 4ED5^[^ ^47^ ^]^ and 6GD3,^[^ ^48^ ^]^ respectively. C) Simulated flexible Brownian dynamics solutions for HuR_106‐326_ sampling ANP32A_230‐249_ and ANP32B_232‐251_ peptides. Encounters ensembles simulated for HuR_106‐326_ with ANP32A_230‐249_ (*left panels*) and ANP32B_232‐251_ (*right panels*). Representations of the peptide structures show ribbons colored in black for the best‐fitted solution provided by CYANA validation for each construct. The most represented conformations in the solutions given by BD simulations are colored in orchid for ANP32A_230‐249_ and light blue for ANP32B_232‐251_. Mass centers of HuR_106‐326_ corresponding to the 50000 registered complexes for each simulation are shown as cyan spheres.

The LCARs of ANP32A and ANP32B are implicated in the binding of these proteins with multiple targets, such as the polymerase and nucleoprotein of the influenza A virus or the RNA‐binding protein HuR.^[^ ^3^, ^20^, ^21^ ^]^ As the Pro241 substitution alters their affinity for C*c*, we explored the possibility that the interaction with other ligands could also be affected. To test our hypothesis, we studied HuR interactions with the ANP32A_230‐249_ and ANP32B_232‐251_ peptides. We designed an HuR construct containing the RRM domains 2 and 3, separated by the hinge region of HuR (HuR_106‐326_), which interacts with ANP32 proteins.^[^ ^3^ ^]^ We then performed ITC assays with HuR_106‐326_ and the ANP32A_230‐249_ or ANP32B_232‐251_ peptides (**Figure** **6**A). Similar to C*c*’s affinity, HuR's affinity was ca. 5‐fold higher for ANP32A_230‐249_ than for ANP32B_232‐251_ at low ionic strength, suggesting that the open conformation of the ANP32A peptide enhances interactions with ANP32 target proteins through the C‐terminal LCAR. ITC results indicate that two molecules of each ANP32 peptide bind to HuR_106‐326_, with the interaction driven by enthalpy (Table S2, Supporting Information).

This finding perfectly matches with rigid‐body/docking *ab‐initio* BD simulations, with HuR_106‐326_ as the target of each ANP32 peptide. Both ANP32A_230‐249_ and ANP32B_232‐251_ peptides mainly explored the hinge region of HuR (Figure 6B), in agreement with previous reports.^[^ ^3^, ^22^ ^]^ While the HuR/ANP32B complex showed two preferred, most delimited binding sites for the ANP32B_232‐251_ peptide (in agreement with the stoichiometry HuR:ANP32B of 1:2 inferred by ITC), the ANP32A_230‐249_ peptide widely sampled most of the positively‐charged HuR_106‐326_. These differences in modes of interaction resembled those described for the C*c*/ANP32 complexes mentioned above, with the ANP32B_232‐251_ peptide exhibiting a most limited flanking of HuR, probably accounting for a reduced interacting surface. Both interactions were characterized by a negative enthalpy due to long‐range electrostatic interactions during HuR‐involving complexes formation under low ionic strength. The highly positively‐charged HuR hinge—with a p*I* of ca. 11.5 and several His residues susceptible of protonation and deprotonation at physiological pH—makes electrostatics key in HuR/ANP32 adducts (Figure 6B).

Flexible BDs of HuR_106‐326_ and the ANP32A_230‐249_ or ANP32B_232‐251_ peptide revealed that HuR explores the C‐end of both peptides, although the contacts with the ANP32A_230‐249_ peptide map to a broader region (Figure 6C). Thus, the open conformation found in the ANP32A C‐terminal region might represent a binding favorable arrangement, influencing the protein contacts and subsequent process regulation.

## 3 Discussion

In this work, we demonstrate the importance of punctual modifications in IDDs for the dynamics and function of the proteins, using the Pro/Thr evolution in ANP32A/ANP32B as an example.

Molecular models of the C‐terminal regions of ANP32A and ANP32B substantially differ in their structure and dynamics (Figure 2A). The presence of a Pro residue near the ANP32A NLS restraints local, transient contacts between the opposite charged stretches of the LCAR. Our SASA and RMSF data demonstrate that the ANP32A_230‐249_ peptide presents a more open, extended, and solvent‐accessible arrangement as compared to the ANP32B_232‐252_ peptide (Figure 2D,E).

Experiments with C*c*, a protein target of ANP32 LCAR, confirmed the functional consequences of the Pro substitution. First, we showed that C*c* can recognize the LCAR of ANP32A under DNA damage, since C*c* is relocated to the nucleus under such conditions (Figure 3; Figure S2, Supporting Information). However, at low ionic strength, C*c* affinity is higher for ANP32A (*K*_D_ *ca*. 3.6 µM) (Figure 3C) than for ANP32B (*K*_D_ *ca*. 9.5 µM),^[^ ^31^ ^]^ and differences in the enthalpic and entropic terms indicate alterations in the recognition mode that correlate with conformational propensities (Table 1). These differences become more evident for C*c*’s interactions with the ANP32A_230‐249_ and ANP32B_232‐252_ peptides (Table 2). In particular, the interaction between C*c* is entropically‐driven for ANP32A peptide but enthalpically‐driven for the ANP32B peptide. Moreover, binding of C*c* to the ANP32A_230‐249_ peptide is weakened by the P241T single‐mutation (*K*_D_: 190 µM). This mutation is enough to fit the thermodynamic parameters into an enthalpy‐driven profile, whereas the double‐mutation Q235E/P241T achieves a very similar thermodynamic profile to the one of ANP32B_232‐251_ (Table 2 and Figure S4, Supporting Information). These results support our hypothesis that Pro241 determines a specific local conformation within the surroundings of the ANP32A NLS, which impedes the electrostatic contacts between the basic NLS and the acidic residues of the LCAR. This disruption favors the conformational freedom of the ANP32A LCAR, allowing it to sample the C*c* surface in a more flexible manner, which explains the relevance of the entropic term. The P241T mutation allows the NLS to bury in the surrounding environment, due to its contacts with LCAR acidic residues, thus explaining the enthalpic contribution. In the double‐mutated ANP32A_230‐249_ Q235E/P241T peptide, the additional negative charge flanking the NLS enhances these transient electrostatic contacts.

Our results from the NMR titration experiments of C*c* with the ANP32A_230‐249_ or ANP32B_232‐252_ peptide, along with both flexible and NMR‐restraint docking BD simulations, reinforce that ANP32A recognizes a broader surface area of C*c*. NMR assays showed that C*c* uses not only the area surrounding the heme group to interact with ANP32A—which is likewise the case for ANP32B—but also some residues located at the opposite side (such as Asp2 and Glu89) (Figure 4C).^[^ ^31^ ^]^ This spread pattern on C*c* surface, along with the dynamics of the hemeprotein in complex with ANP32A_230‐249_ inferred from NMR relaxation measurements (Figure 5), point out that C*c* and ANP32A form a *fuzzy* ensemble, more structurally heterogeneous and with a higher degree of plasticity than the C*c*:ANP32B complex.^[^ ^31^ ^]^ Given the basic character of the NLS (Lys and Arg residues), it seems plausible that it will interact with the acidic Asp2 and Glu89 of C*c*. The rest of the ANP32A LCAR would remain flexible in solution, contacting with C*c* at the heme‐surrounding and the opposite surface areas. This kind of interaction can be described as a flanking model. The flanking model assumes that the IDDs use short recognition motifs surrounded by flexible stretches. Upon complex formation, the flanking sequences might maintain their flexibility and conformational variability, thereby allowing additional contacts with the corresponding protein partner.^[^ ^43^, ^44^, ^45^ ^]^ The flanking model also fits the overall entropic gain during the formation of ANP32A:C*c* complex. The entropy gain during the disorder‐to‐order transition of IDDs is a paradox that can be explained by their extended binding interfaces. While certain motifs become relatively rigid upon binding (with local entropy loss), the regions linking them remain flexible (with global entropy gain). Thus, the desolvation entropy gain counterbalances the conformational local entropy loss of the IDD.^[^ ^41^, ^45^ ^]^ In contrast, the transient contacts into the LCAR of ANP32B restrict its conformation, altering the entropic terms of ANP32B interactions with C*c*.

The Pro‐to‐Thr substitution might affect not only the interactions of ANP32 proteins with C*c* but also with other binding partners. ITC assays revealed that the enthalpically‐driven binding of the ANP32 LCAR peptides with HuR_106‐326_ is stronger for ANP32A_230‐249_ than for ANP32B_232‐252_, proving substantial differences in the interaction with both ANP32 proteins (Figure 6A and Table S2, Supporting Information). BD simulations corroborated the broader mapping of the ANP32A_230‐249_ peptide as compared with the ANP32B_232‐252_ peptide (Figures 4C and 6B). ANP32 proteins are responsible for the HuR shuttling from the nucleus to the cytoplasm upon heat shock, where it carries out its main functions.^[^ ^22^, ^49^ ^]^ Moreover, interactions of ANP32 proteins with HuR also modulates its RNA binding capacity.^[^ ^3^ ^]^ Therefore, the subtle differences in the mode of interaction of each ANP32 protein with HuR could represent a fine‐tuning regulation of such processes.

Altogether, these data agree with different binding modes of ANP32A and ANP32B to their common protein targets C*c* and HuR, due to a distinct conformational arrangement of their LCARs. C*c* and HuR represent two examples of the possible consequences of the Pro‐to‐Thr substitution at the C‐terminal IDD. It is highly probable that other protein targets have also a preference for a specific LCAR conformation. This is also the case of the influenza A virus nucleoprotein and polymerase. ANP32 proteins are essential in viral RNA replication during infection, since they ensure the co‐replicative assembly of RNA into the ribonucleoprotein complexes. For that purpose, ANP32 proteins regulate the dimerization of the polymerase, and they interact with the nucleoprotein. The implication of the LCAR has been recently described for both mechanisms, which suggests possible differences in the recognition of the influenza A virus nucleoprotein and polymerase by the ANP32A and ANP32B proteins.^[^ ^20^, ^21^ ^]^ Indeed, the avian influenza virus polymerase seems to selectively interact with the N‐terminal region of the LCAR of ANP32B but not that of ANP32A,^[^ ^50^ ^]^ which corroborates the importance of small differences in the IDD, as the sequence identity of the structured LRR is larger than 70%.

## 4 Conclusion

The ANP32A and ANP32B proteins regulate several important functions in the cell. In this work, we demonstrated the importance of a Pro substitution in mammalian evolution, which has a big influence in the binding of ANP32 proteins with their targets. Therefore, we propose that the Pro‐to‐Thr substitution (from ANP32A to ANP32B) might have been stabilized as it provides a mechanism that specifically modulates protein functionality at a tissue‐specific level.

## 5 Experimental Section

### DNA Constructs

The genes encoding the different ANP32A constructs for transfection in mammalian cells were cloned into the pcDNA3.1 or pcDNA3 vectors using the In‐Fusion HD EcoDry Cloning Kit (Clontech) according to the manufacturer's instructions. Details on the procedure were given in the Supporting Information Appendix.

### ANP32 Proteins C‐Terminal Synthetic Peptides

Synthetic peptides containing the C‐end stretch of ANP32A and ANP32B LCAR domains were ordered to Genecust (France). The peptide sequences were as follows:

ANP32A_230‐249_: EEERGQKRKREPEDEGEDDD;

ANP32A_230‐249_ P241T: EEERGQKRKRETEDEGEDDD;

ANP32A_230‐249_ Q235E/P241T: EEERGEKRKRETEDEGEDDD;

ANP32B_232‐251_: EEGGKGEKRKRETDDEGEDD.

### Cell Cultures and Transfection

HEK293T (human embryonic kidney 293T) and Heltog cells were cultured in a humidified atmosphere at 5% CO_2_ and 37 °C and grown in Dulbecco's Modified Eagle Medium (Sigma Aldrich) supplemented with 10% of heat‐inactivated fetal bovine serum (FBS), 2 mM L‐glutamine, 100 U mL^−1^ penicillin and 100 µg mL^−1^ streptomycin. More details on the procedure were given in the Supporting Information Appendix.

### Subcellular Localization Assays

After 1 h or 4 h of CPT treatment, nuclei were stained by incubating cells with 1 µg mL^−1^ Hoechst (Sigma‐Aldrich) for 10 min at 37 °C. Cells were then rinsed in pre‐warmed PBS at 37 °C to remove media traces, fixed by immersing the coverslips in a 4% formaldehyde (Sigma‐Aldrich) solution prepared in PBS for 10 min at room temperature, and then washed in pre‐warmed PBS at 37 °C. Cells were dried by incubating the coverslips in absolute ethanol for 2 min. Afterward, coverslips were mounted into glass slides using N‐propyl gallate (Sigma‐Aldrich), to preserve the fluorescence, and sealed with nail polish. Images were obtained in a Zeiss LSM 7 DUO confocal microscope using a 63× oil objective.

### Western Blot and Pulldown Assays

Pulldown assays were performed as previously described.^[^ ^31^ ^]^ Briefly, transfected HEK293T cells were resuspended in a lysis buffer containing 10 mM Tricine‐NaOH pH 8.5, 1 mM PMSF, and cOmplete protease inhibitors (Roche). Afterward, cells were lysed by sonication (10 s, 10% amplitude, on ice), and the resulting debris was discarded after 15 min of centrifugation at 13300 rpm and 4 °C. More details on the procedure were given in the Supporting Information Appendix.

### Nuclear Magnetic Resonance

2D ^1^H‐^1^H Total Correlation Spectroscopy (TOCSY) and nuclear Overhauser effect spectroscopy (NOESY) spectra recorded on ANP32 peptides were measured at 10 °C on a Bruker Avance‐III 700 MHz spectrometer. Samples were prepared by resuspending the peptide stock in 10 mM sodium phosphate buffer pH 6.3, along with 10% D_2_O to adjust the lock signal. Due to the acidic nature of the peptides, the pH was measured and corrected before each measurement. Specific NMR parameters were set: d9 value (evolution time) in TOCSY was 80 ms; d8 value (mixing time) in NOESY was 400 ms. Details on the procedure were given in the Supporting Information Appendix.

### ANP32 Peptide Model Calculation

ANP32A_230‐249_ and ANP32B_232‐251_ peptide models were calculated using CYANA 2.1 (L.A. Systems, Inc. Japan). NOESY‐derived distance restraints were applied to the calculation with 100 initial structures annealed to 20 final structures. Distance violations in the final models were revised and erased if present in more than 15 structures of the final structure ensemble. The process was repeated until no distance was violated in the final models. Further solvent‐accessible surface area and atomic fluctuations analyses were performed using CPPTRAJ.^[^ ^51^ ^]^

### Calculation of the Secondary Structural Propensity of ANP32 Peptides

The H_N_ and H_α_ chemical shifts of ANP32A_230‐249_ and ANP32B_232‐251_ peptides were used to estimate the secondary structure propensity at residue level. This was carried out by using different algorithms: i) the neighbor corrected structure propensity calculator (ncSPC),^[^ ^52^ ^]^ which bases its calculation on the ncIDP random coil library and adds an additional weighting procedure that accounts for the backbone conformational sensitivity of each amino acid type; and ii) the CSI 3.0 web server, which uses backbone chemical shifts to identify up to eleven different types of secondary structures.^[^ ^53^ ^]^

### Calculations of Brownian Dynamics

Brownian dynamics (BD) computations were carried out using the SDA‐flex 7.2.4 software.^[^ ^54^, ^55^ ^]^ For each ANP32A_230‐249_ and ANP32B_232‐251_, the top ten validated structural models were used as input for flexible BD simulations. For C*c* computation, the 2N9I PBD entry was used.^[^ ^56^ ^]^ For HuR_106‐326_, the structural model was built using MODELLER.^[^ ^57^ ^]^ Details on the procedures were given in the Supporting Information Appendix.

### Isothermal Titration Calorimetry

Isothermal titration calorimetry (ITC) experiments were performed using a Nano ITC Low Volume instrument (TA Instruments, USA). The reference cell was filled with distilled water. Titration experiments between reduced C*c* and ANP32A or ANP32A_1‐167_ consisted of 17 injections of 2.91 µL of a 400 µM reduced C*c* solution into the sample cell containing 50 µM ANP32A construct. Both C*c* and ANP32A proteins were dialyzed against 10 mM sodium phosphate buffer pH 7.4. Assays performed at moderate ionic strength used the same experimental setup, but proteins were dialyzed against 20 mM sodium phosphate pH 7.4 with 50 mM NaCl. Assays using ANP32A_230‐249_ or ANP32B_232‐251_ peptides were performed by titrating 1 mM peptide solution onto 110 or 150 µM reduced C*c* solutions, respectively, or onto 100 µM HuR_106‐326_ solution, following the same injection configuration as described for ANP32A proteins. C*c* and HuR_106‐326_ solutions were dialyzed against 10 mM sodium phosphate buffer pH 6.3; peptides were dissolved in the same solution. The stirring speed was set to 200 rpm for HuR_106‐326_ titration experiments and to 300 rpm for C*c* titration experiments, to ensure cell homogeneity. ANP32A, ANP32A_230‐249_ and ANP32B_232‐251_ titration data were analyzed using Origin 7.0 (OriginLab), employing a single‐ligand binding site model or a two‐ligand binding sites model. In the last case, no binding cooperativity was observed. ANP32A_1‐167_ titration data was processed and analyzed using NanoAnalyze software (TA instruments).

## Conflict of Interest

The authors declare no conflict of interest.

## Author Contributions

M.A.R., I.D.M. performed conceptualization. B.B.J., A.B.U., F.R.R., M.A.C.C., Al.V.C., Ad.V.C., L.C.G., M.A.R., I.D.M. performed methodology. B.B.J., A.B.U., F.R.R., M.A.C.C., Al.V.C., L.C.G. performed investigation. Ad.V.C., L.C.G., M.A.R., I.D.M. performed supervision. F.R.R., M.A.C.C., Al.V.C., I.D.M. wrote — the original draft. B.B.J., A.B.U., M.A.C.C., Ad.V.C., L.C.G., M.A.R., I.D.M. wrote — review & edited original draft.

## Supporting information



Supporting Information

## Data Availability

All data are available in the main text or the supplementary materials.
