# Supplementary material for: Evolutionary Pro‐To‐Thr Mutation in the Intrinsically Disordered Domain of ANP32 Family Members Modulates Their Target Binding Modes
Source: Adv Sci (Weinh). 2025 Jan 31;12(12):2415566. doi: 10.1002/advs.202415566 (PMC11948038; doi:10.1002/advs.202415566)
Supplement: Supplementary file 1 — Supporting Information [file ADVS-12-2415566-s001.docx]

Supporting Information

**Evolutionary Pro-to-Thr mutation in the intrinsically disordered domain of ANP32 family members modulates their target binding modes**

*Blanca Baños-Jaime^1,#^, Ana B. Uceda-Mayo^1,#^, Francisco Rivero-Rodríguez^1,#^, Miguel Á. Casado-Combreras^1^, Alejandro Velázquez-Cruz^1^, Adrián Velázquez-Campoy^2,3,4,5^, Laura Corrales-Guerrero^1,*^, Miguel A. De la Rosa^1^ and Irene Díaz-Moreno^1,*^*

^1^Institute for Chemical Research (IIQ), Scientific Research Center “Isla de la Cartuja” (cicCartuja), University of Seville-CSIC, Seville, Spain.

^2^Institute for Biocomputation and Physic of Complex Systems (BIFI), Joint Unit GBsC-CSIC-BIFI, University of Zaragoza, Zaragoza, Spain.

^3^Departament of Biochemistry and Molecular and Cellular Biology, University of Zaragoza, Zaragoza, Spain.

^4^Institute for Health Research of Aragón (IIS Aragon), Zaragoza, Spain.

^5^Centre for Biomedical Research Network of Hepatic and Digestive Diseases (CIBERehd), Madrid, Spain.

^#^Equal contribution

*Corresponding authors. E-mail addresses: [laucorge@us.es](mailto:laucorge@us.es) (L. Corrales-Guerrero) & [idiazmoreno@us.es](mailto:idiazmoreno@us.es) (I. Díaz-Moreno)

## This PDF file includes:

- Supporting Figures S1 to S5
- Supporting Tables S1 to S2
- Supporting Information References

# Supporting Information Text

# Extended Materials and Methods

# Nuclear magnetic resonance

2D ^1^H-^1^H Total Correlation Spectroscopy (TOCSY) and nuclear Overhauser effect spectroscopy (NOESY) spectra recorded on ANP32 peptides were measured at 10 ºC on a Bruker Avance-III 700 MHz spectrometer. Samples were prepared by resuspending the peptide stock in 10 mM sodium phosphate buffer pH 6.3, along with 10% D_2_O to adjust the lock signal. Due to the acidic nature of the peptides, pH was measured and corrected before each measurement. Specific NMR parameter were set: d9 value (evolution time) in TOCSY was 80 ms; d8 value (mixing time) in NOESY was 400 ms.

In ANP32A_230-249_, Pro241 was assigned in both *cis* and *trans* isomers. From the intensity of resonances, it can be stated that the *cis* configuration significantly prevails over the trans configuration (*cis*:*trans* signal intensity ratio in percentage is ca. 95:5). Note that this study refers exclusively to *cis*-Pro241 ANP32A when "ANP32A" is used.

NMR titrations of 30 μM ^15^N reduced C*c* with ^14^N ANP32A constructs were followed by 1D ^1^H and 2D ^1^H-^15^N heteronuclear single quantum correlation (HSQC) spectra on a Bruker Avance-III 600 MHz equipped with a cryoprobe at 25 ºC. ^15^N-labelled reduced C*c* (in 10 mM sodium phosphate buffer, pH 7.4) was titrated with increasing amounts of ^14^N ANP32A dialyzed against the same buffer. Reduced ^15^N C*c* and ^14^N ANP32A_1-167_ construct were titrated in 5 mM sodium phosphate buffer pH 6.4. To maintain the redox state of C*c* and to adjust the lock signal on the NMR spectrometer, 0.1 mM of sodium ascorbate and 10% of D_2_O were added, respectively. Data were acquired and processed using TopSpin NMR 3.5pl7 software (Bruker).

Titration experiments between C*c* and ANP32A_230-249_ and ANP32B_232-251_ peptides were followed by 1D ^1^H and 2D ^1^H-^15^N HSQC spectra on a Bruker Avance-III 500 MHz and 600 MHz, respectively, both equipped with a cryoprobe at 25 ºC. ^15^N-labelled, reduced C*c* (50 μM in 10 mM sodium phosphate buffer, pH 6.3) was titrated with increasing amounts of ^14^N ANP32 peptides (Genecust), which were resuspended in the same buffer. D_2_O (10%) and sodium ascorbates were added to adjust the lock signal on the NMR spectrometer and to ensure the redox state of C*c*, respectively.

Line broadening and chemical-shift perturbation analysis were performed using the Sparky 3 NMR assignment tool (T.D. Goddard and D.G. Kneller, University of California San Francisco, USA). Assignment of ANP32A_230-249_ and ANP32B_232-251_ peptides was performed using NMRFAM-SPARKY software distribution (National Magnetic Resonance Facility at Madison) and further imported to CCPNMR ^[1]^, whereby distance restraints were calculated using its built-in module.

The difference (each difference calculated as follows: ΔX=X_C_*_c_*__ANP32A_- X_C_*_c_*__ANP32B_) in ^15^N relaxation parameters (longitudinal relaxation rate (*R*_1_), transversal relaxation rate (*R*_2_), and heteronuclear ^15^N{^1^H} NOE) were obtained from experiments recorded at 500 MHz ^1^H frequency at 25 °C for the complexes formed between reduced C*c* and ANP32A_230-249_ or ANP32B_232-251_ peptides. Samples containing ^15^N-labelled C*c* (500 µM) in the presence of ^14^N ANP32A peptide (323 µM) or ^14^N ANP32B peptide (1 mM) were prepared in 10 mM sodium phosphate buffer pH 6.3. D_2_O (5%) and sodium ascorbate were added to adjust the lock signal on the NMR spectrometer and to ensure the redox state of C*c*, respectively. The *R*_1_ parameter was calculated using 10 delays ranging from 10 to 1000 ms, and the *R*_2_ parameter was calculated using 10 delays ranging from 16.96 to 118.72 ms. The ^15^N{^1^H} NOE was determined by recording spectra with and without a 4.2 s long proton saturation period. The spectra were processed using NMRPipe and analyzed with NMRDraw ^[2]^. Peak intensities were extracted from each spectrum, with *R*_1_ and *R*_2_ parameters for each sample obtained by fitting the data to an exponential decay function: $I_{t}=I_{0}e^{({-tR}_{1,2})}$, where *I_0_* represents the peak intensity at time 0 and *I_t_* represents the intensity at time t. The *R*_1_ and *R*_2_ parameters were used to estimate the rotational correlation time (τ_c_) of the complexes using TENSOR 2.0 ^[3].^

# ANP32 peptide model calculation

ANP32A_230-249_ and ANP32B_232-251_ peptide models were calculated using CYANA 2.1 (L.A. Systems Inc., Japan). NOESY-derived distance restraints were applied to the calculation with 100 initial structures annealed to 20 final structures. Distance violations in the final models were revised and erased if present in more than 15 structures of the final structure ensemble. The process was repeated until no distance was violated in the final models. Further solvent-accessible surface area and atomic fluctuations analyses were performed using CPPTRAJ ^[4]^.

# Cell cultures

HEK293T (human embryonic kidney 293T) and Heltog cells (HeLa cells constitutively expressing the C*c* gene fused to green fluorescent protein, [GFP]) were cultured in a humidified atmosphere at 5% CO_2_ and 37 ºC and grown in Dulbecco’s Modified Eagle’s Medium (Sigma Aldrich) supplemented with 10% of heat-inactivated fetal bovine serum, 2 mM L-glutamine, 100 U/mL penicillin and 100 µg/mL streptomycin.

For calcium phosphate transfection, 2.5 × 10^6^ HEK293T cells were cultured in 150 mm diameter plates. After 72 h of growth, media was refreshed prior to transfection. For subcellular localization assays; 50,000 Heltog cells were grown over coverslips placed in 15.6 mm well plates for 24 h before transfection. DNA damage was then induced by replacing media for one containing 20 µM of camptothecin (CPT) and incubating cells for 1 h or 4 h.

# Cell transfection

HEK293T cells were transfected using calcium phosphate, as previously described ^[5]^. Cells were harvested at 48 h post-transfection using a scraper and 5 mL of chilled phosphate buffer saline (PBS) to retrieve detached cells. Media and cellular debris were removed by aspiration after 5 min of centrifugation at 1,500 rpm. Cells were washed with 5 mL of chilled PBS to remove the remaining media traces and centrifuged for 5 min; the supernatant was then removed, and cells were resuspended in a specific lysis buffer for each different assay.

For subcellular localization assays, Heltog cells were transfected with the plasmid pcDNA3-ANP32A-mCherry using Lipofectamine 2000 (Invitrogen) following the manufacturer’s instructions.

# Subcellular localization assays

After 1 or 4 h of CPT treatment, nuclei were stained by incubating cells with 1 µg/mL Hoechst (Sigma-Aldrich) for 10 min at 37 ºC. Cells were then rinsed in pre-warmed PBS at 37 ºC to remove media traces, fixed by immersing the coverslips in a 4% formaldehyde (Sigma-Aldrich) solution prepared in PBS for 10 min at room temperature, and then washed in pre-warmed PBS at 37 ºC. Cells were dried by incubating the coverslips in absolute ethanol for 2 min. Afterwards, coverslips were mounted into glass slides using N-propyl gallate (Sigma-Aldrich), to preserve the fluorescence, and sealed with nail polish. Images were obtained in a Zeiss LSM 7 DUO confocal microscope using a 63× oil objective.

# Pulldown assays

Pulldown assays were performed as previously described ^[6]^. Briefly, transfected HEK293T cells were resuspended in a lysis buffer containing 10 mM Tricine-NaOH pH 8.5, 1 mM PMSF and cOmplete™ protease inhibitors (Roche). Afterwards, cells were lysed by sonication (10 s, 10% amplitude, on ice), and the resulting debris was discarded after 15 min of centrifugation at 13,300 rpm and 4 ºC.

C*c* pulldown assays were carried out by incubating cell extracts with 100 µg of recombinant C*c* for 16 h at 4 ºC in batch. A carboxymethylcellulose matrix (Whatman) was added, and the mixture was incubated for 30 min at 4 ºC in batch. To remove possible non-specific binding, the matrix was washed 3 times with 1 mL of washing buffer containing 10 mM Tricine-NaOH pH 8.5, 20 mM NaCl, 1 mM PMSF and cOmplete™ protease inhibitors. The C*c*:ANP32A complex was eluted from the matrix with an elution buffer containing 10 mM Tricine-NaOH pH 8.5, 360 mM NaCl, 1 mM PMSF, and cOmplete™ protease inhibitors. Results were checked by Western blot analysis, using antibodies against *c*-myc tag (EMD Millipore, #05-724) and C*c* (obtained by immunizing male rabbits with full-length recombinant C*c*).

Cell extracts from HEK293T transfected with an empty vector were processed as described above as a negative control to discern non-specific binding.

# Coimmunoprecipitation assays

HEK293T cells were transfected with a plasmid encoding ANP32A/b-c-myc. 24 h after transfection, cells were collected for lysates preparation in buffer containing 20 mM Tris-HCl pH 7.5, 100 mM NaCl, 0.5% NP40, 0.1 mM PMSF and cOmplete™. Cells were lysed by sonication and precleared by incubation with sepharose-A beads (GE Healthcare, GE17-5280-01) for 1 h at 4 ºC. The lysates were then incubated with anti-c-myc (see Pull-down assays) over night at 4 ºC and subsequently with sepharose-A beads (GE Healthcare, GE17-5280-01) for 1 h at room temperature with rotation. Following 6 washes with PBS, the samples were resuspended in SDS loading buffer and analyzed by western blotting.

# DNA constructs

The genes encoding the different ANP32A constructs for transfection in mammalian cells were cloned into the pcDNA3.1 or pcDNA3 vectors using the In-Fusion HD EcoDry Cloning Kit (Clontech) according to the manufacturer’s instructions.

ANP32A and ANP32A_1-167_ genes were cloned into the pcDNA3.1 vector, which was linearized by polymerase chain reaction (PCR) using the following primers: 5’-GAGCAGAAACTCATCTCTGAA-3’ as the forward primer and 5’-GGATCCGAGCTCGGTACCAAG-3’ as the reverse primer. The ANP32A coding sequence was amplified using 5’-ACCGAGCTCGGATCCATGGAGATGGGCAGACGGATT-3’ as a forward primer and 5’-GATGAGTTTCTGCTCGTCATCATCTTCTCCCTCATC-3’ as a reverse primer, and the ANP32A_1-167_ construct was amplified using the same forward primer as for ANP32A and 5’-GATGAGTTTCTGCTCCAGGCCCTCCACGTAGCCCTC-3’ as a reverse primer.

The DNA coding for ANP32A was cloned into the pcDNA3-mCherry and pEGFP-N1 mammalian expression vectors. pcDNA3-mCherry was linearized using the following primers: 5’-GGGCGCGCCATGGTGAGCAAGGGC-3’ as a forward primer and 5’-AAGGCCGGCCTGATCCGAGCTCGG-3’ as a reverse primer. ANP32A coding sequence was amplified using the 5’-GATCAGGCCGGCCTTATGGAGATGGGCAGACGGATT-3’ as a forward primer and 5’-CACCATGGCGCGCCCGTCATCATCTTCTCCCTCATC-3’ as a reverse primer. The gene of ANP32B was inserted in the pEGFP-N1 vector using the In-Fusion HD EcoDry Cloning Kit (Clontech). Forward and reverse primers 5’-TAAGCGGCCGCGACTCTA-3’ and 5’-TCATGTCCATTCCGCCCAGATCCTCTTCA-GAGATGAGTTTCTGCTCCATGAATTCGAAGCTTGAGCTCGA-3’ were used to linearize pEGFP-N1 vector while inserting a N-terminal *c*-myc tag. Primer pair 5’-TCTGGGCGGAATGGACATGAAGAGGAGGATC-3’ and 5’-TCTAGAGTCGCGGCCG-CTTAATCATCTTCTCCTTCATCATCTGT-3’ was used to amplify ANP32B coding sequence. ANP32A gene was inserted into the same plasmid with the In-Fusion HD EcoDry Cloning Kit (Clontech) using the primer pair 5’-TAAGCGGCCGCGACTCTA-3’ and 5’-CATTCCGCCCAGATCCTCT-3’ to open the pEGFP-N1 plasmid carrying the N-terminal *c-*myc tag, and the primer pair 5’-GATCTGGGCGGAATGGAGATGGGCAGACGGATTC-3’ and 5’-AGTCGCGGCCGCTTAGTCATCATCTTCTCCCTCATCTTC-3’ to amplify ANP32A coding sequence.

The coding sequence for HuR_106-326_ was inserted into a pGEX-2T plasmid using the In-Fusion HD EcoDry Cloning Kit (Clontech). Primers pair 5’-CTGACTGACGATCTGCCTCG-3’ and 5’-GGCTGCTGGAATTCCACCAC-3’ were used to open the plasmid pGEX-2T, and primers 5’-GTGGTGGAATTCCAGCAGCCGCCAACTTGTACATCAGCGG-3’ and 5’-CGAGGCAGATCGTCAGTCAGTTATTTGTGGGACTTGTTGG-3' were used to amplify HuR_106-326_ gene from a pGEX-4T-2 plasmid carrying HuR full-length ^[5]^. After ligation, plasmid was open avoiding GST-tag sequence, by using primers 5'-TCGGATCTGGTTCCGCGTG-3' and 5'-GAATACTGTTTCCTGTGTGAAATT-GTTATCCG-3'. A sequence coding for a 6xHis-tag and an MBP-tag, amplified with the oligonucleotide pair 5'-CAGGAAACAGTATTCATGGCACACCATCACC-ACCATCACAGCAGC-3' and 5'-CGGAACCAGATCCGAACCGCTGCTAGT-CTGCGCGTC-3' from pOPINM plasmid, was inserted using the In-Fusion HD EcoDry Cloning Kit (Clontech).

# Western blot assays

Samples were resolved by sodium dodecyl sulphate-polyacrylamide gel electrophoresis (SDS-PAGE) at 15% acrylamide. Polyvinylidene fluoride (PVDF) membranes (EMD Millipore) were used to transfer the protein samples with a Mini Trans-Blot electrophoretic transfer cell (Bio-Rad). Non-specific signals were avoided by incubating the membrane with 5% non-fat dry milk in PBS with 0.1% Tween-20 (TPBS).

Immunoblotting was performed using specific primary antibodies against targeted proteins. The primary antibodies used were: anti-*c*-myc tag clone 4A6 (EMD Millipore, #05-724), and anti-human C*c* serum (obtained by immunizing male rabbits with recombinant C*c*). Two different HRP-conjugated secondary antibodies were used for detection, depending on the primary antibody host (Mouse IgG, Sigma Aldrich, A9044; Rabbit IgG, Sigma Aldrich, A0545). Immunoreactive bands were detected using Amersham ECL Plus Western Blotting Detection Reagents (GE Healthcare Life Sciences).

# Protein expression and purification

C*c* was expressed in *E. coli* BL21 (DE3) and purified as previously described ^[8]^.

The ANP32A and ANP32A_1-167_ recombinant proteins were also expressed in *E. coli* BL21 (DE3) cells. A single colony was selected from a LB-agar plate supplemented with 100 mg/mL kanamycin after electroporation of a vial of *E. coli* BL21 (DE3) cells in the presence of 100 ng of pET28a(+)-ANP32A or pET28a(+)-ANP32A_1-167_. The colony was used to inoculate 50 mL of Luria Bertani (LB) media pre-cultures supplemented with 100 mg/mL kanamycin. After 16 h of incubation at 37 ºC with 150 rpm shaking, the pre-cultures were used to inoculate 2 L of LB media, also supplemented with kanamycin, at a 1:1,000 ratio. Cultures were grown at 37 ºC; once the optical density at 600 nm reached 0.4-0.6 value, 1 mM isopropyl-b-D-thiogalactopyranoside (IPTG) was added to induce protein expression. Bacteria expressing ANP32A were grown at 16 ºC for 16 h, whereas those expressing ANP32A_1-167_ were grown at 37 ºC.

The HuR_106-326_ protein was expressed in *E. coli* BL21 (DE3) cells in LB medium supplemented with 100 mg/mL ampicillin for 60 h at 12 ºC and 150 rpm after induction with 1 mM IPTG.

Cells were harvested by centrifugation (5 min at 6,000 rpm) and resuspended in a lysis buffer containing 20 mM Tris-HCl pH 8.0, 100 mM NaCl, 1 mM DTT, 1 mM PMSF, 0.2 mg/mL lysozyme, 0.02 mg/mL DNase, and cOmplete™ protease inhibitors (Roche). Cells were lysed by sonication (cycles of 30 s at 40% amplitude, 60 s of rest, 3 min total time or 10 s at 35% amplitude, 30 s of rest, 4 min total time, always on ice). Cellular debris were removed by centrifuging the samples for 30 min at 20,000 rpm and 4 ºC. The supernatant was subsequently loaded onto a Ni-NTA matrix (Generon), previously equilibrated with lysis buffer, and incubated in batch for 1 h at 4 ºC. Recombinant proteins were eluted with a non-continuous imidazole gradient and their purity was further checked by SDS-PAGE. Fractions containing pure ANP32A, ANP32A_1-167_ or HuR_106-326_ were merged and dialyzed against buffer without imidazole for 24 h at 4 ºC. For ANP32A, impurities were removed with an additional size-exclusion purification step, using a Superdex 200 10/300 GL (GE Healthcare) column and 10 mM sodium phosphate pH 7.4 as the running buffer. Purity was later checked by SDS-PAGE and proteins were concentrated using 3 or 30 kDa M.W.C.O. Pierce Protein Concentrators (Thermo Fisher Scientific).

# Isothermal titration calorimetry

ITC experiments were performed using a Nano ITC Low Volume instrument (TA Instruments, USA). The reference cell was filled with distilled water. Titration experiments between reduced C*c* and ANP32A or ANP32A_1-167_ consisted of 17 injections of 2.91 μL of a 400 μM reduced C*c* solution into the sample cell containing 50 μM ANP32A construct. Both C*c* and ANP32A proteins were dialyzed against 10 mM sodium phosphate buffer pH 7.4. Assays performed at moderate ionic strength used the same experimental setup, but proteins were dialyzed against 20 mM sodium phosphate pH 7.4 with 50 mM NaCl. Assays using ANP32A_230-249_ and ANP32B_232-251_ peptides were performed by titrating 1 mM peptide solution onto 110 or 150 μM reduced C*c* solutions, respectively, or onto 100 μM HuR_106-326_ solution, following the same injection configuration as described for ANP32A proteins. C*c* and HuR_106-326_ solutions were dialyzed against 10 mM sodium phosphate buffer pH 6.3; peptides were dissolved in the same solution. The stirring speed was set to 200 rpm for HuR_106-326_ titration experiments and to 300 rpm for C*c* titration experiments, to ensure cell homogeneity. ANP32A, ANP32A_230-249_ and ANP32B_232-251_ titration data was analyzed using Origin 7.0 (OriginLab), employing a single ligand binding site model or a two ligand binding sites model. In the last case, no binding cooperativity was observed. ANP3_2A1-167_ titration data were processed and analyzed using NanoAnalyze software (TA instruments).

# Biolayer Interferometry

Bio-layer interferometry (BLI) measurements were performed on an Octet RED96e System (Sartorius, USA) using Dip and Read Streptavidin Biosensors (SA). All assays were performed at 25 ºC in buffer containing 20 mM sodium phosphate pH 7.4 with 50 mM NaCl with agitation at 1,000 rpm. 0.5 μM biotinylated ANP32A or ANP32B were immobilized on biosensors and probed with different concentrations of C*c*. The association step was followed for 1,200 s, and dissociation (by dilution in binding buffer) was measured for 500 s. To determine the equilibrium dissociation constant (*K*_D_), the maximal wavelength shifts measured at the end of the association phases were plotted against the corresponding protein concentrations. The data were then subjected to curve fitting in Origin 7.0 using an equation for specific binding: Y = B_max_ * X / (*K*_D_ + X).

# Calculations of Brownian dynamics

Brownian dynamics (BD) computations were carried out using the SDA-flex 7.2.4 software ^[9,10]^. For each ANP32A_230-249_ and ANP32B_232-251_, the top ten validated structural models were used as input for flexible BD simulations. For C*c* computation, the 2N9I PBD entry was used ^[11]^. For HuR_106-326_, the structural model was built using MODELLER ^[12]^. PQR files were obtained using the TLEAP module of the AMBER 18 package ^[13]^ and force fields electrostatic, desolvation, and hydrophobic grids for every conformer were generated with the ECM module of SDA-flex 7.2.4 ^[14]^ and APBS 3.0 ^[15]^. Translational and rotational diffusion constants were calculated using the script ARO ^[16]^, on VMD ^[17]^.

Computations of 25,000 diffusion trajectories were performed for flexible BD simulations at 15 mM ionic strength.

*Ab initio* docking simulations of both ANP32_A230-249_ or ANP32B_232-251_ onto C*c* or HuR_106-326_ were performed by computing each ANP32 construct as the most represented conformer among the solutions provided by the corresponding flexible simulation. In this case, 10,000 trajectories were computed. Every complex was recorded except when the root mean square deviation (RMSD) value was lower than 2 Å with respect to a previous one.

NMR-driven docking simulations of both ANP32_A230-249_ or ANP32B_232-251_ onto C*c* were performed under similar conditions under experimental restrictions derived from the results of the corresponding NMR titrations. C*c* residues considered for restrictions were those showing Δδ_AVG_  ≥  0.01 ppm and a solvent exposed area (SEA) larger than 50%. SEA was calculated with the NACCESS software ^[18]^, basing on the Lee and Richards method ^[19]^. Reaction criteria were defined as < 5-Å contacts between all possible electron donor-acceptor pairs between N atoms of Arg, Lys and Gly side-chains, O atoms of Asp, Glu and Thr side-chains and C-termini, and those of Gly backbone amides. Complexes were registered when they fulfilled at least two reaction criteria separated by at least 5 Å.

Representations were made using UCSF Chimera 1.15 ^[20]^.

# Supporting Figures


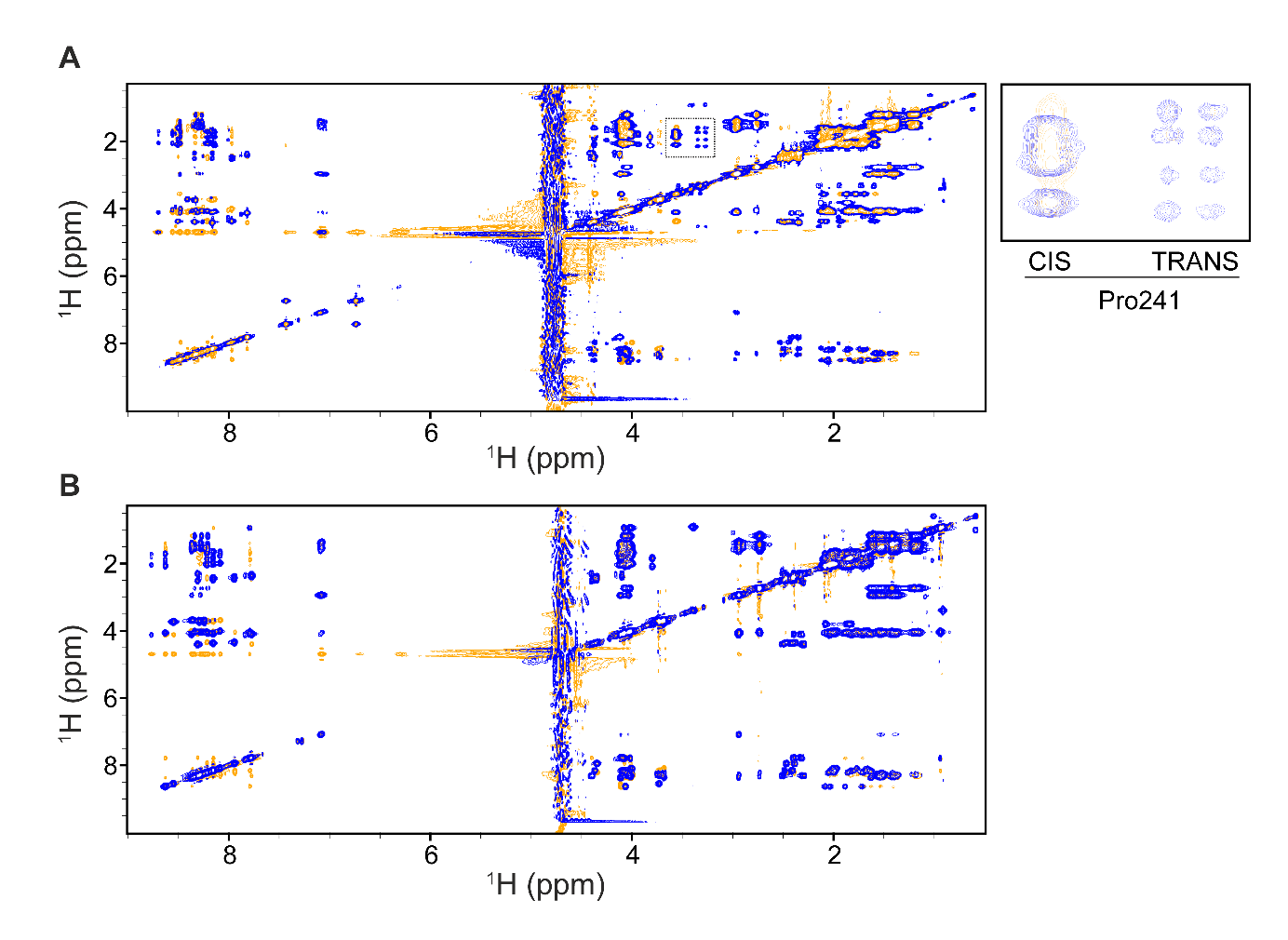


**Figure S1**. **2D ^1^H-^1^H TOCSY and NOESY spectra acquired for WT ANP32A_230-249_ or WT ANP32B_232-251_ peptides**. Superimposition of the 2D ^1^H-^1^H TOCSY (blue) and NOESY spectra (orange) of ANP32A_230-249_ (**A**) or ANP32B_232-251_ (**B**) peptides at 10 ºC. In panel A, the inset details the *cis* and *trans* conformation of ANP32A_230-249_ Pro241.


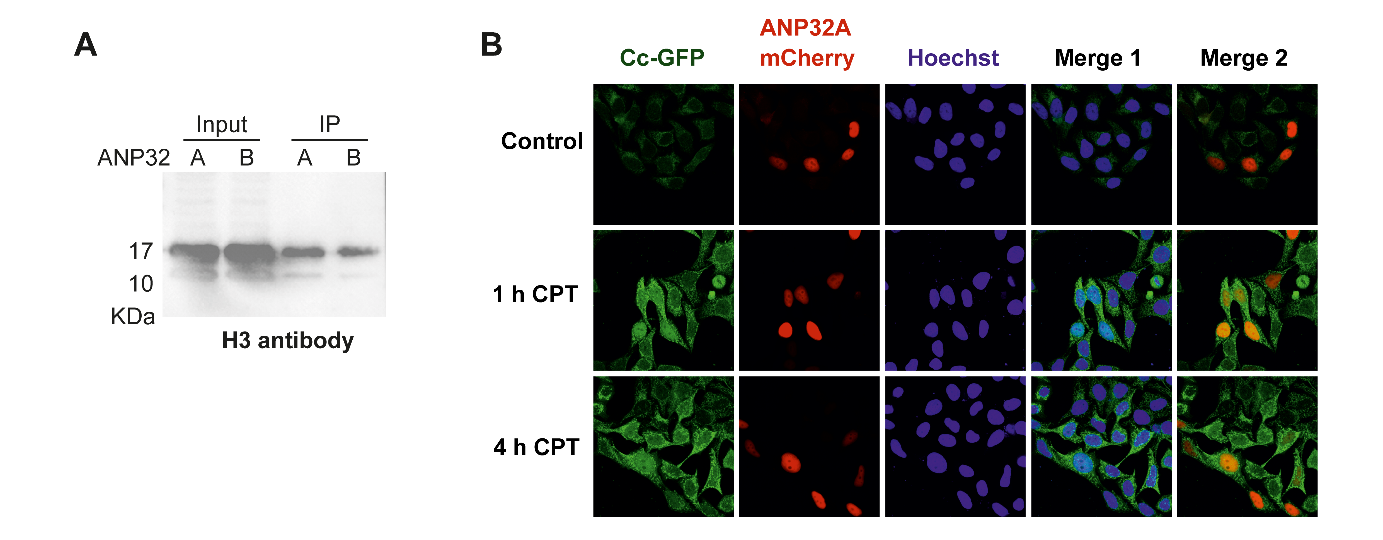


**Figure S2**. **Analysis of ANP32 proteins functionality in the cell**. (**A**) Coimmunoprecipitation experiment. HeLa cell lysates containing *c*-myc-ANP32A/B were immobilized on Sepharose beads conjugated with anti-*c*-myc antibodies, and tested against histone H3 antibodies by western-blot analysis. Shown is a representative image from three experiments. (**B**) Subcellular co-localization of chimeric C*c*-GFP (green channel) and ANP32A-mCherry (red channel) in Heltog cells under homeostatic conditions (Control) and upon CPT-induced DNA damage for 1 h and 4 h. Nuclei are stained with Hoechst. Merge 1 corresponds to the overlay images of C*c*-GFP (green) and Hoechst (blue), and merge 2, to the overlay images of C*c*-GFP (green) and ANP32A-mCherry (red) fluorescence.

**
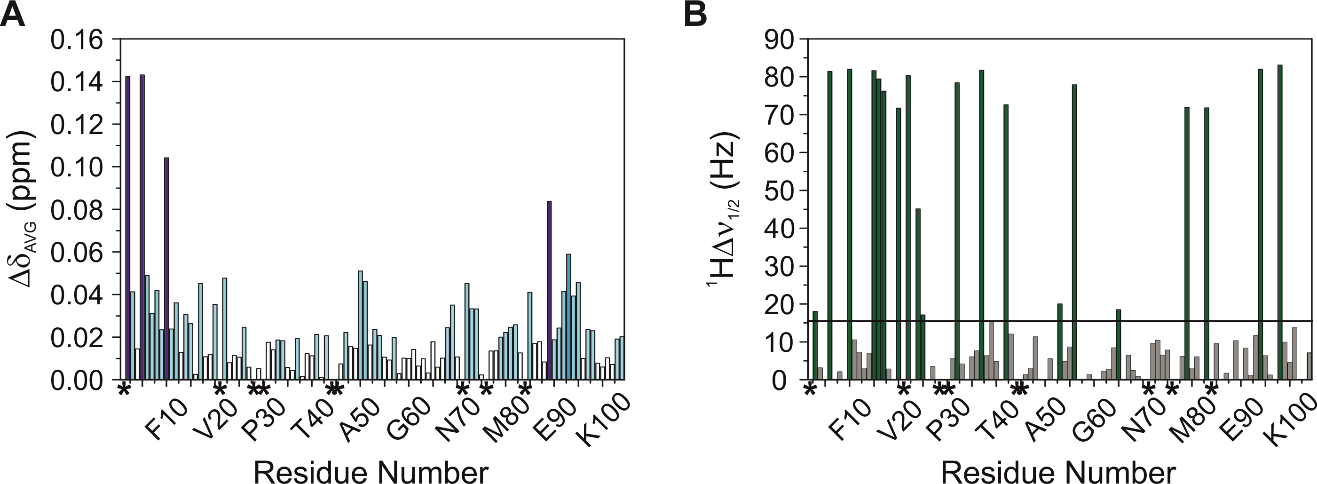
**

**Figure S3. NMR titrations of ^15^N-labeled cytochrome *c* with ANP32A.** (**A**) Plot of Δδ_AVG_ of ^15^N-labeled C*c* in the presence ANP32A at a 1:1 molar ratio as a function of residue number. Bars are colored according to a Δδ_AVG_ gradient ranging from white (Δδ_AVG_ = 0.0 ppm) to dark blue (Δδ_AVG_ > 0.075 ppm). (**B**) ^1^H line-width differences (^1^HΔν_1/2_) between free and C*c* bound with ANP32A at a 1:1 molar ratio as a function of residue number. Green and gray bars correspond to residues exhibiting a broadening larger or lower than the threshold, respectively. This threshold value (15.96 Hz) corresponds to the average background broadening plus two SDs. For **A** and **B**, asterisks (*) indicate prolines and non-assigned residues.


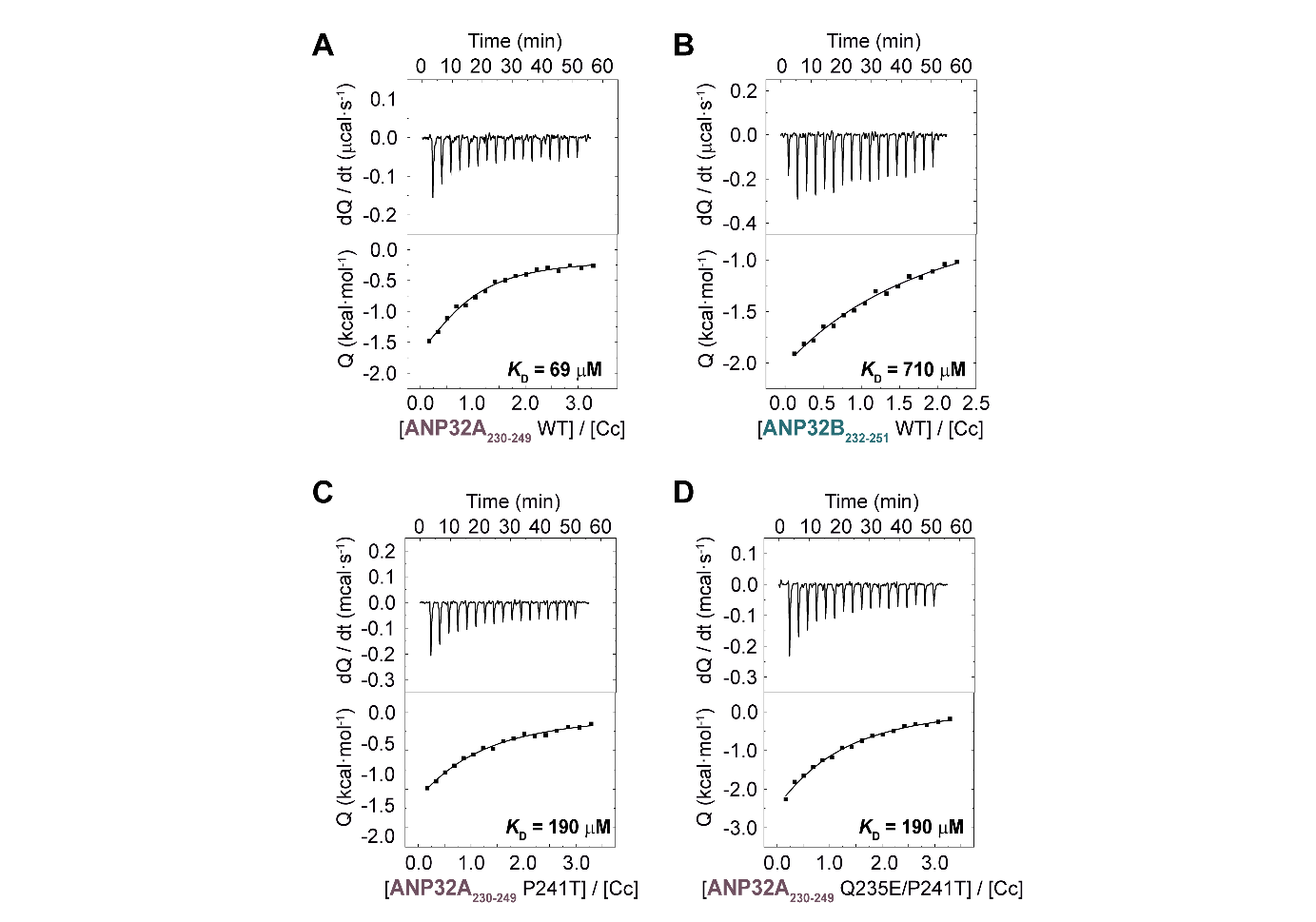


**Figure S4. ITC binding experiments of cytochrome *c*-ANP32A/B LCAR peptide complexes.** Titrations of C*c* are shown with WT ANP32A_230-249_ (**A**), WT ANP32B_232-251_ (**B**), ANP32A_230-249_ P241T (**C**), or ANP32A_230-249_ Q235E/P241T (**D**). Thermograms and binding isotherms are shown in the *upper* and *lower* panels, respectively.

**
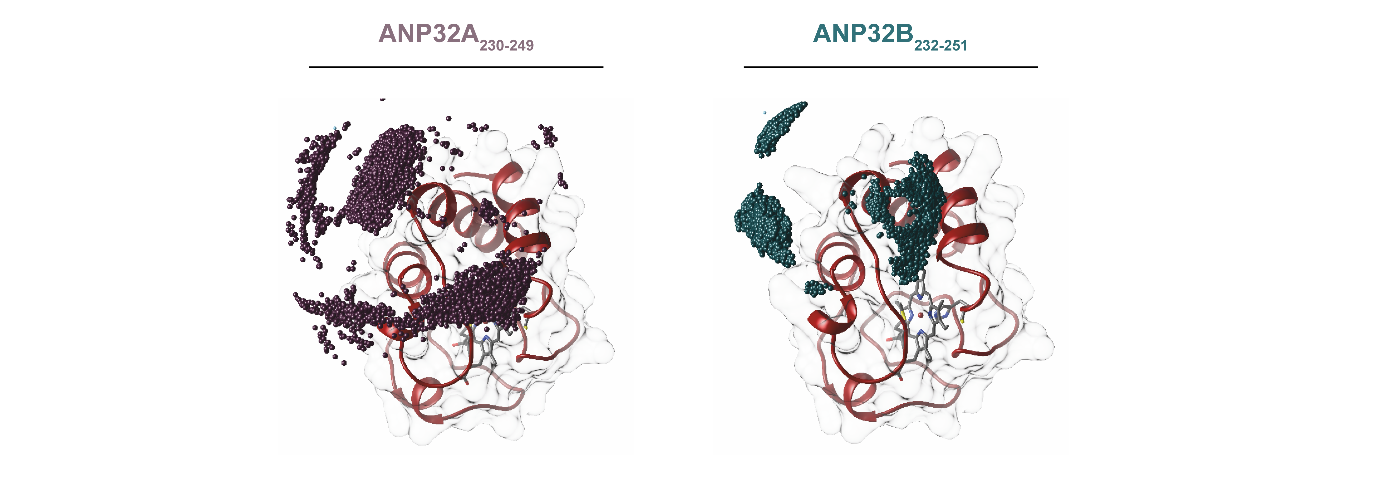
**

**Figure S5. *Ab initio* Brownian Dynamics solutions for ANP32A_230-249_ and ANP32B_232-251_ complexes with C*c*.** Mass centers distributions of ANP32A_230-249_ (*left panels*) or ANP32B_232-251_ (*right panels*), representing the 10,000 lowest energy conformations of both complexes with C*c*. Mass centers are shown as violet spheres for ANP32A_230-249_ and blue spheres for ANP32B_232-251_. Ribbon and surface areas of C*c* (PDB entry 2N9I) are shown in red; the heme group, in light green.

# Supporting Tables

**Table S1. Structural statistics for the calculations of ANP32A_230-249_ and ANP32B_232-251_ peptides. Statistics were calculated for the 20 lowest energy structures predicted of each peptide by CYANA**

| NMR restraints | ANP32A_230-249_ | ANP32B_232-251_ |
| --- | --- | --- |
| Distance restraints | 193 | 187 |
| Restraints statistics^a^ |  |  |
| Distance violations > 0.0 Å | 0 | 0 |
| Pairwise RMSD of residues 1-20 in Å^b^*^,^*^c^ |  |  |
| Backbone N, Ca, CO | 3.53 ± 0.74 | 2.63 ± 0.75 |
| Heavy atoms | 4.80 ± 0.77 | 3.77 ± 0.75 |
| Ramachandran plot^c^ |  |  |
| Most favoured regions (%) | 40.7 | 28.6 |
| Additional allowed regions (%) | 51.7 | 62.5 |
| Generously allowed regions (%) | 7.3 | 8.9 |
| Disallowed regions (%) | 0.3 | 0.0 |
| CYANA Target Function | 0.15 ± 0.007 | 0.01 ± 0.003 |

^a^ Violations are only reported when present in five or more structures.

^b^ Coordinate precision is given as the average pair-wise Cartesian coordinate root mean square deviations over the ensemble.

^c^ Values obtained from the PROCHECK-NMR analysis ^[21]^ by using the Protein Structure Validation Server (PSVS) ^[22]^.

**Table S2. Thermodynamic parameters of HuR_106-326_:ANP32A/B peptide complexes**

| **Protein complex** | ***K*_D_**  (μM) | **Δ*G***  (kcal/mol) | **Δ*H***  (kcal/mol) | –***T*Δ*S***  (kcal/mol) | ***n*** |
| --- | --- | --- | --- | --- | --- |
| **HuR_106-326_:ANP32A_230-249_** | 120 | -5.3 | -8.4 | 3.1 | 1.8 |
| **HuR_106-326_:ANP32B_232-251_** | 550 | -4.4 | -8.8 | 4.4 | 1.9 |

Thermodynamic parameters for the interaction of ANP32A/B peptides with HuR_106-326_. Equilibrium dissociation constant (*K*_D_), enthalpy (**Δ***H*), Gibbs free energy (**Δ***G*), entropic (–*T***Δ***S*), and reaction stoichiometry (*n*) are shown. Relative errors: *K*_D_ 20%, **Δ***G* 0.1 kcal/mol, **Δ***H* and *-T***Δ***S* 0.4 kcal/mol, and *n* 0.03.

# Supporting Information References

[1] S. P. Skinner, R. H. Fogh, W. Boucher, T. J. Ragan, L. G. Mureddu, G. W. Vuister, *J. Biomol. NMR* **2016**, *66*, 111.

[2] F. Delaglio, S Grzesiek, G. W. Vuister, G. Zhu, J. Pfeifer, A. Bax, *J. Biomol. NMR* **1995**, *6*, 277.

[3] P. Dosset, J. C. Hus, M. Blackledge, D. Marion, *J. Biomol. NMR* 2000, *16*, 23.

[4] D. R. Roe, T. E. Cheatham, *J. Chem. Theory Comput.* **2013**, *9*, 3084.

[5] R. E. Kingston, C. A. Chen, J. K. Rose, *Curr. Protoc. Mol. Biol.* **2003**, *63*, Unit 9.1.

[6] F. Rivero-Rodríguez, A. Díaz-Quintana, A. Velázquez-Cruz, K. González-Arzola, M. P. Gavilan, A. Velázquez-Campoy, R. M. Ríos, M. A. De la Rosa, I. Díaz-Moreno, *Redox Biol.* **2021**, *43*.

[7] R. M. Scheiba, Á. Aroca, I. Díaz-Moreno, *Eur. Biophys. J.* **2012**, *41*, 597.

[8] B. Moreno-Beltrán, I. Díaz-Moreno, K. González-Arzola, A. Guerra-Castellano, A. Velázquez-Campoy, M. A. De La Rosa, A. Díaz-Quintana, *FEBS Lett.* **2015**, *589*, 476.

[9] R. R. Gabdoulline, R. C. Wade, *Methods A Companion to Methods Enzymol.* **1998**, *14*, 329.

[10] M. Martinez, N. J. Bruce, J. Romanowska, D. B. Kokh, M. Ozboyaci, X. Yu, M. A. Öztürk, S. Richter, R. C. Wade, *J. Comput. Chem.* **2015**, *36*, 1631.

[11] M. Imai, T. Saio, H. Kumeta, T. Uchida, F. Inagaki, K. Ishimori, *Biochem. Biophys. Res. Commun.* **2016**, *469*, 978.

[12] B. Webb, A. Sali, *Methods Mol. Biol.* **2017**, *1654*, 39.

[13] D.A. Case, I.Y. Ben-Shalom, S.R. Brozell, D.S Cerutti, T.E. III Cheatham, V.W.D. Cruzerio, T.A. Darden, R.E. Duke, D. Ghoreishin, M.K. Gilson, et al., *Univ. California, San Fr.* **2018**.

[14] R. R. Gabdoulline, R. C. Wade, **1996**.

[15] N. A. Baker, D. Sept, S. Joseph, M. J. Holst, J. A. McCammon, *Proc. Natl. Acad. Sci. U. S. A.* **2001**, *98*, 10037.

[16] I. Díaz-Moreno, F. J. Muñoz-López, E. Frutos-Beltrán, M. A. De la Rosa, A. Díaz-Quintana, *Bioelectrochemistry* **2009**, *77*, 43.

[17] W. Humphrey, A. Dalke, K. Schulten, *J. Mol. Graph.* **1996**, *14*, 33.

[18] S. J. Hubbard, J. M. Thornton, *NACCESS, Computer Program, Department of Biochemistry and Molecular biology, University College London*, **1993**.

[19] B. Lee, F. M. Richards, *J. Mol. Biol.* **1971**, *55*, 379.

[20] E. F. Pettersen, T. D. Goddard, C. C. Huang, G. S. Couch, D. M. Greenblatt, E. C. Meng, T. E. Ferrin, *J. Comput. Chem.* **2004**, *25*, 1605.

[21] R. A. Laskowski, J. A. C. Rullmann, M. W. MacArthur, R. Kaptein, J. M. Thornton, *J. Biomol. NMR* **1996**, *8*, 477.

[22] A. Bhattacharya, R. Tejero, G. T. Montelione, *Proteins Struct. Funct. Genet.* **2007**, *66*, 778.
